# *PDK4* as a metabolic biomarker of chronic hydrocephalus

**DOI:** 10.3389/fgene.2026.1780231

**Published:** 2026-03-19

**Authors:** Robbie Clarke, Payton Villers, Chloe Bills, Michaela Rice, Madison Higgins, Chan Lee, Prabir Patra, Peter H.U. Lee, Abhay Moghekar, Joon W. Shim

**Affiliations:** 1 Department of Biomedical and Electrical Engineering, Marshall University, Huntington, WV, United States; 2 Department of Anesthesiology, Perioperative, and Pain Medicine, Brigham and Women’s Hospital and Harvard Medical School, Boston, MA, United States; 3 Department of Cardiothoracic Surgery, Southcoast Health, Fall River, MA, United States; 4 Department of Pathology and Laboratory Medicine, Brown University, Providence, RI, United States; 5 Department of Neurology, Johns Hopkins School of Medicine, Baltimore, MD, United States

**Keywords:** caudate nucleus (CN), hydrocephalus, *PDK4*, RNA biomarker, RNA-seq

## Abstract

**Background:**

Chronic hydrocephalus (CH) is a heterogeneous neurological disorder characterized by persistent ventricular enlargement and neurovascular dysfunction in the aging brain. Despite its clinical relevance, genetically anchored RNA biomarkers reflecting CH-associated metabolic and stress-related pathology remain poorly defined.

**Methods:**

We performed bulk RNA sequencing of postmortem caudate nucleus tissue from individuals with CH and age-matched neurologically normal controls. Disease-associated transcriptional programs were identified using principal component analysis (PCA), unsupervised hierarchical clustering, and gene set enrichment analysis (GSEA). Key candidate transcripts were validated by RT-PCR. Comparative genomic analyses across mouse, rat, pig, and human genomes examined transcript length, chromosomal positioning, and nucleotide composition.

**Results:**

PCA of the top 1,000 most variable transcripts demonstrated robust separation between CH and controls. Analysis of transcripts ranked 1,001–2,000 independently reproduced disease segregation, indicating distinct transcriptional programs. GSEA revealed significant enrichment of xenobiotic metabolism and oxidative stress pathways, with pyruvate dehydrogenase kinase 4 (*PDK4*) emerging as the top-ranked gene among ∼40,000 transcripts. RT-PCR confirmed robust *PDK4* upregulation. Comparative genomics showed conserved transcript length but increased telomeric proximity and A+T content in humans.

**Conclusion:**

PDK4: is identified as a prominent RNA marker of chronic hydrocephalus in the elderly, providing a neurogenomic foundation for future fluid-based RNA biomarker development.

## Introduction

Chronic hydrocephalus (CH) is a neurological condition characterized by the accumulation of cerebrospinal fluid (CSF) in the cerebral ventricles, leading to ventricular enlargement, increased intracranial pressure, and progressive neuronal dysfunction (Dombrowski et al., 2008; de Beer and Scheltens, 2016; Buscemi et al., 2024; Gallina et al., 2024). Although CH predominantly affects aging individuals (de Beer and Scheltens, 2016) in the type of idiopathic normal pressure hydrocephalus (Brautigam et al., 2019), its underlying molecular mechanisms related to specific biomarkers remain poorly understood. In contrast, histopathological biomarkers such as amyloid beta (aβ), phosphorylated tau, and phosphorylated alpha-synuclein are well-established in Alzheimer’s disease (AD) and Parkinson’s disease (PD) (Braak et al., 1993; Braak and Braak, 1997; Braak et al., 2003; Ganguly et al., 2017). However, the lack of similarly defined biomarkers in hydrocephalus presents a significant knowledge gap, hindering the development of effective diagnostic tools and targeted therapies. This underscores the need for comprehensive investigations into the underlying pathogenesis of hydrocephalus.

Previous studies on inflammatory biomarkers of hydrocephalus across all ages underscore their significance; however, they have primarily focused on interleukins and tumor necrosis factor (*TNF*) in cerebrospinal fluid (CSF), rather than in the brain parenchyma or tissue (Lattke et al., 2012; Xu et al., 2012; Oria et al., 2019; Goulding et al., 2020; Harris et al., 2021; Garcia-Bonilla et al., 2022; Holste et al., 2022; Dutra et al., 2023; Fang et al., 2024). It has been reported that periventricular reactive gliosis and/or microgliosis, marked by increased Glial Fibrillary Acidic Protein (*GFAP*) or Ionized Calcium-Binding Adapter Molecule 1 (*IBA1*) expression, indicates that glial cells, including astrocytes and microglia, play a central role in neuroinflammation associated with hydrocephalus. (Ulfig et al., 2004; Deren et al., 2010; Tang et al., 2022; Zhang et al., 2022; Zhu et al., 2022; Luikku et al., 2024; Mayo et al., 2024). Although periventricular gliosis and microgliosis serve as clear histopathological hallmarks of neuroinflammation in hydrocephalus, an advanced technique like bulk RNA-seq may not effectively capture their significance due to the dilution of region-specific cellular changes within the broader tissue transcriptome, the heterogeneous composition of brain samples, and the limitations of RNA expression in reflecting cellular activation states (Rao et al., 2018). Given this, recent studies have begun to highlight *RELA* (p65) activation as a key indicator of *NF-κB* involvement in hydrocephalus, suggesting that classical *NF-κB* signaling may be critical in the inflammatory response and disease progression (Lattke et al., 2012; Lin et al., 2022; Xu et al., 2024). Activation of inhibitor of nuclear factor kappa-B kinase subunit beta (*IKK2*) in astrocytes has been shown to impair ependymal ciliogenesis, leading to hydrocephalus in mice (Lattke et al., 2012). Here, ikk2 activation evokes p50, p65 (=*RELA*), and *C-REL* but not *RELB*.

Pyruvate dehydrogenase kinase 4 (*PDK4*) is a key regulator of cellular energy metabolism that modulates the flux of pyruvate into the tricarboxylic acid cycle by inhibiting the pyruvate dehydrogenase complex (Park et al., 2018). *PDK4* is highly responsive to metabolic stressors, including hypoxia, oxidative stress, nutrient deprivation, and inflammatory signaling, and its induction promotes a metabolic shift away from mitochondrial oxidative phosphorylation toward glycolytic or lipid-based energy utilization (Thoudam et al., 2019; Sugiura et al., 2022; Deng et al., 2025). Dysregulation of *PDK4* has been implicated in systemic metabolic disorders, ischemic injury, and neurodegenerative conditions, reflecting its role as a stress-adaptive metabolic switch (Yin et al., 2021). In the central nervous system, altered *PDK4* expression has been observed in settings of impaired perfusion and mitochondrial dysfunction, suggesting that it may serve as a sensitive indicator of chronic neurovascular and metabolic stress (Zhao et al., 2020; Kim et al., 2024). Given the sustained alterations in cerebrospinal fluid dynamics, perfusion, and oxygen delivery characteristic of chronic hydrocephalus (Leinonen et al., 2017; Zhang et al., 2024; Back et al., 2025), investigation of *PDK4* expression in affected brain regions may provide critical insight into disease-associated metabolic reprogramming and identify a candidate RNA biomarker (Hanson et al., 2018) reflective of underlying pathophysiology.

Recent advancements in transcriptomics have provided powerful tools to elucidate the molecular and genetic changes associated with various neurological disorders, including CH (Barrett et al., 2024; Shim et al., 2024). Studies focusing on the caudate nucleus—a brain region integral to motor control, learning, and memory—are particularly relevant, given its vulnerability in CH (Luciano et al., 2001; Cirak et al., 2020). The vascular alterations in the caudate nucleus observed in the previous dog model (capillary density increase after initial decline) suggest a hypoxia-driven response to CH (Luciano et al., 2001). The caudate nucleus is also a site where oxidative stress, hypoxia, and neuroinflammation are believed to converge, contributing to neuronal damage and disease progression. However, the specific genetic and pathway-level alterations occurring in this region in CH remain largely unexplored.

In this study, we used whole transcriptome RNA sequencing (RNA-Seq) to analyze gene expression profiles in the caudate nucleus of individuals with CH and age-matched controls. By integrating principal component analysis (PCA), hierarchical clustering, and pathway enrichment analyses, we aimed to characterize the genetic landscape of CH, with particular emphasis on pathways identified through RNA-seq–based bioinformatic analyses and subsequently validated by RT-PCR, alongside genomic analyses examining telomeric proximity and nucleotide composition associated with elevated mutation rates in selected marker genes. The findings from this study provide a comprehensive overview of the transcriptomic alterations in CH, offering new insights into its molecular pathogenesis and potential avenues for therapeutic intervention.

## Results

### Global transcriptomic reprogramming in chronic hydrocephalus reveals distinct primary and secondary gene programs

**FIGURE 1:**
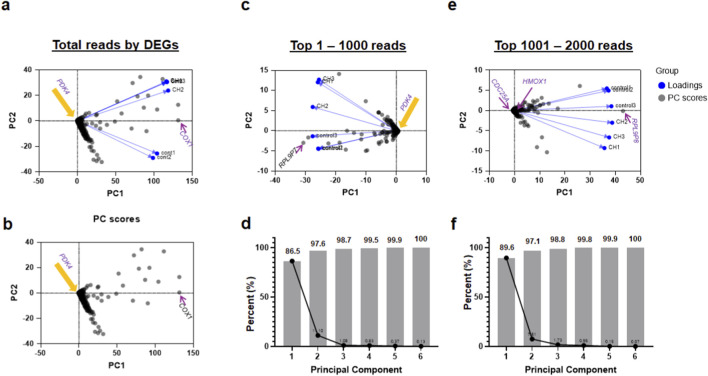
Principal component analysis reveals distinct primary and secondary transcriptional programs associated with chronic hydrocephalus. Plots illustrating the use of dimensionality reduction to evaluate sample variance and diagnostic segregation across different data scopes, from total transcriptomic reads to high-significance gene subsets. **(a)** Biplot of PCA analysis on total reads (∼40,000): Principal component analysis performed on the entire dataset highlights global sample distribution. As indicated by the orange arrow, *PDK4* is localized near the origin (0,0), suggesting it is not a primary driver of global variance at this comprehensive scale. In contrast, genes like *COX1* (purple arrow) located further along the PC1 axis contribute more significantly to the global variance. **(b)** PC scores plot: A simplified version of the biplot in panel **(a)**, focusing exclusively on the spatial distribution of individual samples to reduce plot complexity. The orange arrow confirms that *PDK4* remains at the mathematical center of the global score distribution. **(c)** Top 1–1,000 by DEG analysis: Analysis of the most significant 1,000 differentially expressed genes (DEGs) reveals improved segregation between control and CH groups. While markers like *RPL9P7* (purple arrow) pull the variance toward the control groups, *PDK4* (orange arrow) remains near the origin, indicating its expression acts as a stable representative for the group mean rather than an outlier driver within this specific PCA projection. **(d)** Proportion of Variance (Top 1–1,000): Scree plot showing the percentage of variance explained by each principal component. PC1 and PC2 combined account for the vast majority of variance (97.6%), validating the 2D projections. **(e)** Next top 1,001–2000 reads: PCA of the secondary tier of significant reads demonstrates a unique pattern while maintaining diagnostic segregation. At this tier, genes such as *CDC25A*, *HMOX1*, and *RPL9P8* (purple arrows) emerge as more prominent drivers of variance compared to the primary set. **(f)** Proportion of Variance (Top 1,001–2,000): Similar to panel **(d)**, this plot confirms that the primary variance (PC1) remains a dominant driver (89.6%) even within the secondary tier of significant genes.

To determine the primary drivers of transcriptomic variance in the human postmortem caudate nucleus, we performed Principal Component Analysis (PCA) across three tiers of reads based on DEG ranking (Figures 1a–f). Cumulative variance analysis confirmed that the first principal component (PC1) accounted for a substantial majority of the variance, ranging from 86.5% to 89.6% across the different read groups (Figures 1d,f).

Intriguingly, we observed a distinct separation between magnitude of differential expression and contribution to global variance. Although *PDK4* was identified as the top-ranked DEG by the sequencing facility, it localized near the origin (zero) along the PC1 axis in both the total reads and Top 1–1,000 reads analyses (Figures 1a,c).

In contrast, genes such as *COX1*, *RPL9P7*, and *RPL9P8* were consistently located at the far endpoints of the PC1 axis (Figures 1a,c,e). These genes can serve as major loadings that define the PC1 dimension, indicative of the most significant contributors to the variance that distinguishes the control group from the CH cohort. Contrary to this idea, the integration of both RNA-seq datasets (batch 1 and 2) did not provide statistical support for this (*COX1*, *RPL9P7*, and *RPL9P8*) conjecture (Supplementary Figure S5). This divergence underscores that neither the magnitude of fold-change in top-ranked DEGs nor the high-variance genes identified by PCA are, in isolation, sufficient predictors of cross-batch reproducibility.

### Hierarchical clustering and cluster distribution of differentially expressed genes

**FIGURE 2:**
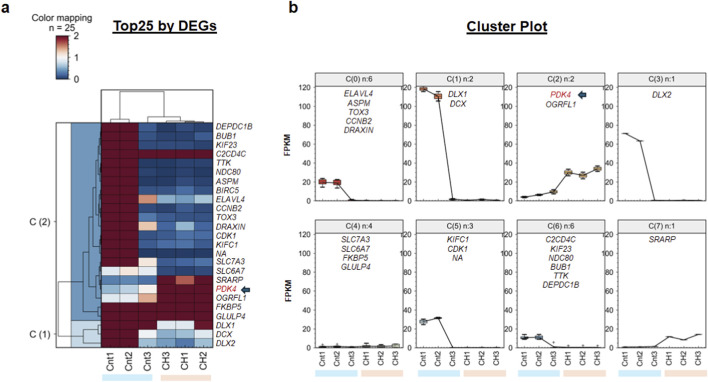
Targeted transcriptomic profiling of the top 25 differentially expressed genes (DEGs) in chronic hydrocephalus. Plots providing a high-resolution analysis of the leading-edge drivers of CH, highlighting the specific pathological signatures and expression trajectories of top-tier markers. **(a)** Heatmap of Top 25 DEGs: Hierarchical clustering reveals distinct contrasts in expression across experimental groups. Cluster C(1) at the bottom of the heatmap identifies genes that are significantly upregulated in CH specimens compared to controls. Within this pathological cluster, *PDK4* (indicated by the red label and arrow) exhibits a clear contrast, shifting from deep blue in controls to vibrant red in CH samples. **(b)** K-means Cluster Trajectories (k = 8): Segmentation of the 25-gene subset into eight distinct expression programs based on FPKM. Cluster C(2) (n = 2): Contains *PDK4* and *OGRFL1*, representing the primary high-magnitude, upward-trending program in the disease state. Cluster C(1) (n = 2): Identifies high-magnitude downregulated drivers, including *DLX1* and *DCX*. Cluster C(0) (n = 6): Captures stable/minor decline trajectories for genes such as *ELAVL4*, *ASPM*, *TOX3*, *CCNB2*, and *DRAXIN*. Cluster C(5) (n = 3): Includes moderately downregulated transcripts such as *KIFC1* and *CDK1*. Cluster C(6) (n = 6): Contains low-level stable genes like *C2CD4C*, *KIF23*, *NDC80*, *BUB1*, *TTK*, and *DEPDC1B*. Specific Trajectories: Secondary upregulated and downregulated markers are further isolated in C(7) (*SRARP*), C(3) (*DLX2*), and C(4) (*SLC7A3*, *SLC6A7*, *FKBP5*, *GLULP4*).

To evaluate the expression patterns of the top differentially expressed genes (DEGs), we performed a hierarchical clustering (HC) analysis, visualized via heatmap (Figure 2a). The dendrogram and color mapping revealed distinct sample-wise and gene-wise groupings, clearly separating the control cohort from the CH samples. This transcriptomic analysis revealed a distinct signature for CH, anchored by significant metabolic and neurodevelopmental drivers involving multiple metabolic, inflammatory, and stress-responsive pathways (Miller et al., 2006; Barrett et al., 2024). PDK4 was identified within a major cluster of genes that are consistently upregulated in the CH group relative to the controls (Figure 2a). This finding aligns with its ranking as a top-tier DEG and suggests its robust activation in the postmortem caudate nucleus under chronic hydrocephalic conditions.

To resolve the most discriminative expression patterns, we performed a high-resolution K-means clustering (k = 8) on the Top 25 DEGs (Figure 2b). The assignment of **PDK4** to Cluster 2 [C(2)] highlights its role as a pivotal metabolic marker within this dataset, and while this specific cluster represents genes with substantial magnitude changes, other identified groupings exhibit distinct expression behaviors. For instance, C(1) and C(0) contain genes that are largely downregulated or exhibit higher variance within the control group, whereas C(6), which includes markers such as ***C2CD4C*** and ***BUB1***, represents genes that maintain high expression across the CH cohort despite significantly lower baseline levels in controls. Ultimately, the robust categorization of **PDK4** into an upregulated hierarchical cluster and its specific localization in C(2) reinforces its candidacy as a reliable biomarker for the metabolic shifts occurring in chronic hydrocephalus.

This cluster (C(2)) specifically isolates genes contributing substantially to the overall disease-associated variance. The stability of ***PDK4*** within this trajectory—also observed in the broader n = 1,000 and n = ∼40,000 datasets—reinforces its role as a primary biomarker for the metabolic reprogramming occurring in the CH brain (Supplementary Figures S1–S4).

### *PDK4*-centered xenobiotic metabolism emerges as a core disease-associated pathway

**FIGURE 3:**
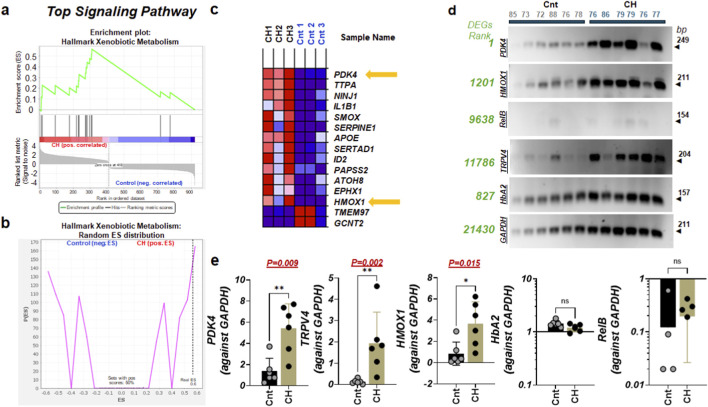
*PDK4* emerged at xenobiotic metabolism **(a)** Gene Set Enrichment Analysis (GSEA) of bulk RNA-sequencing data demonstrates significant positive enrichment of the hallmark xenobiotic metabolism pathway in CH compared to age-matched neurologically normal controls, indicating induction of detoxification and oxidative stress-responsive gene programs in diseased caudate nuclei. The enrichment score (ES) plot (top) shows most xenobiotic metabolism genes ranking toward the CH-associated direction. **(b)** This panel shows the random enrichment score distribution confirming statistical significance of the observed ES. **(c)** Heatmap visualization of leading-edge xenobiotic metabolism genes contributing to the enrichment signal, highlighting consistent upregulation of *PDK4*, *HMOX1*, and associated metabolic/oxidative stress regulators in CH. Samples are clustered by gene expression similarity, revealing clear segregation of CH from control groups. **(d)** Independent validation of selected leading-edge transcripts by RT-PCR analysis confirms elevated mRNA levels of *PDK4* and *HMOX1* in multiple CH biological replicates, consistent with transcriptomic findings. Numerical annotations indicate ranked ordering of genes within the whole-transcriptome variance distribution. **(e)** RT-PCR validation of RNA expression levels in caudate nucleus samples from control and CH groups. Data represents relative quantification against the internal reference, GAPDH. Each dot represents an individual subject. Bars indicate mean ± s.d. Statistical significance was determined using Mann-Whitney test. NS denotes not significant.

Next, gene set enrichment analysis (GSEA) of the bulk RNA-seq data identified significant positive enrichment of the ‘Hallmark Xenobiotic Metabolism’ pathway in CH relative to controls (Figures 3a,b). Leading-edge analysis revealed a consistent upregulation of *PDK4*, *HMOX1*, and related metabolic and oxidative stress–responsive genes (Figure 3c), identifying xenobiotic metabolism as a dominant disease-associated program. This finding aligns with the hierarchical clustering analysis in Figure 2, where *PDK4* was identified within a prominent upregulated gene cluster and specifically categorized into Cluster 2 [C(2)], a grouping defined by sharp, significant increases in expression across the CH cohort. These results suggest that in neurological disorders, altered xenobiotic metabolism often reflects underlying metabolic stress (Dutheil et al., 2008), while the upregulation of these specific processing pathways may represent an activated cellular defense response (Wakabayashi et al., 2006).

Independent RT-PCR validation further substantiated these findings, confirming the previously reported upregulation of *TRPV4* (White et al., 2022) as well as significantly elevated expression of *PDK4* (P = 0.009) and *HMOX1* (P = 0.015) across multiple CH biological replicates (Figures 3d,e; Supplementary Figures S5–S7). The increased band intensities observed for these amplicons were highly consistent with both the RNA-seq–derived normalized expression values and the C(2) trajectory analysis. Collectively, these results establish *PDK4* as a central metabolic stress regulator in the CH caudate nucleus and directly link altered mitochondrial metabolism and oxidative stress responses to the underlying disease pathology.

### Persistent inflammatory, metabolic, and structural dysregulation across the transcriptome

To determine whether disease-associated pathways extend beyond the most highly variable genes, we performed rank-stratified GSEA across successive gene windows encompassing the full ranked transcriptome (Supplementary Figures S1–S4, S8–S19). Analyses of genes ranked 1–1,000 revealed strong enrichment of pathways related to hypoxia, immune activation, inflammatory signaling, cytokine–STAT pathways, epithelial–mesenchymal transition, metabolic regulation, apoptosis, and growth factor signaling (Supplementary Figures S8–S15).

Importantly, many of these pathways remained significantly enriched when analyses were extended to genes ranked 1,001–2,000 (Supplementary Figure S16), indicating that immune, metabolic, and stress-response programs are distributed across broader portions of the transcriptome rather than confined to a narrow subset of highly variable genes. Further extension of the analysis to genes ranked 3,001–4,000 revealed persistent dysregulation of pathways associated with epithelial integrity and cell–cell junction organization (Supplementary Figure S16), suggesting that structural and adhesion-related processes remain affected even among lower-ranked genes.

Additional analyses of subsequent ranked gene intervals (Supplementary Figures S17–S19) further confirmed the robustness and continuity of these disease-associated transcriptional programs across the transcriptome. Across all rank windows examined, leading-edge gene heatmaps consistently demonstrated coordinated expression changes in CH samples relative to controls, reinforcing the conclusion that chronic hydrocephalus is associated with multilayered and persistent transcriptional reprogramming rather than isolated pathway perturbations.

### Evolutionary genomic features suggest increased mutability of *PDK4* and related stress-response genes

**FIGURE 4:**
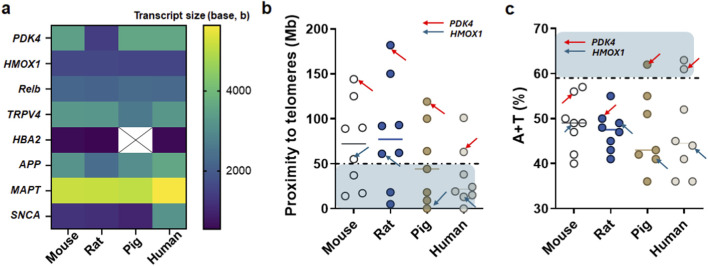
Comparative genomic features underlying relative mutability of *PDK4* and stress-response genes across species. **(a)** Heatmap showing relative ranking of genomic features by transcript sizes (basepair, bp) across representative genes. **(b,c)** Scatter plots illustrating gene-level distributions of telomeric proximity and A+T nucleotide composition, with *PDK4* (red arrows) and *HMOX1* (blue arrows) highlighted to emphasize their relative positioning within mutability-associated genomic space. The blue shaded area in **(b)** represents the genomic proximity to the telomere. We used a critical threshold of 50 million bases (Mb) to define “telomeric proximity,” a value consistent with established parameters in our prior work based on 50 centi-Morgan (cM) ≅ 50 Mb (Nusbaum et al., 2006; McKnight et al., 2021; McKnight et al., 2023). The dashed line indicates this 50 Mb boundary. The shaded area in **(C)** denotes regions where the A+T content exceeds 59% (Nusbaum et al., 2006; McKnight et al., 2021). This threshold was selected because it represents the reported average A+T content across human chromosomes (Nusbaum et al., 2006).

To explore whether *PDK4* exhibits genomic features representing disease-associated dysregulation, we performed quantitative analyses of two factors (McKnight et al., 2021; Barrett et al., 2024) associated with high mutation rates across mouse, rat, pig, and human genomes (Figure 4). *PDK4* transcript length was largely conserved across species, apart from rat, whereas *HBA2* was absent from the annotated pig genome at the genome data viewer (Figure 4a). Analysis of chromosomal positioning revealed a progressive evolutionary migration of *PDK4* and associated transcripts toward telomeric regions, with the distance to the telomere decreasing significantly from mouse to human (Figure 4b). In parallel, A+T nucleotide composition increased across species, with the highest enrichment observed in humans (Figure 4c). Species-specific differences in chromosome length and karyotype organization further highlighted evolutionary remodeling of genomic architecture (Supplementary Figures S20a,b); Supplementary Tables S4–S7). These features suggest that *PDK4* and associated stress-response genes may possess intrinsic genomic properties that facilitate dynamic transcriptional regulation under pathological conditions, contributing to their prominent dysregulation in hydrocephalus of the elderly.

Collectively, these results demonstrate that CH is characterized by robust, multilayered transcriptional reprogramming involving metabolic stress, immune and inflammatory activation, cytokine signaling, and structural remodeling pathways. The persistence of these signatures across multiple rank-stratified gene windows (Supplementary Figures S1–S19) underscores their fundamental role in disease biology rather than stochastic expression variability. PDK4 emerges as a central metabolic node, localized within a permissive genomic architecture that facilitates adaptive transcriptomic responses through evolutionary divergence, linking xenobiotic metabolism to neurovascular and immune dysfunction in the chronically hydrocephalic brain.

## Discussion

In this study, we present a comprehensive transcriptomic analysis of the caudate nucleus in elderly individuals with CH, revealing a robust molecular reprogramming that distinguishes the disease state from age-matched neurologically normal controls. A central and defining finding of this work is the identification of *PDK4* as a primary disease-associated transcript, which, alongside *HMOX1* and the previously identified *TRPV4*, forms a core molecular triad characterizing the CH landscape. While global variance analysis (Figure 1) initially suggested that mitochondrial and ribosomal genes like *COX1* or *RPL9P7* were the dominant drivers of the dataset, these markers failed to maintain statistical significance when integrated across independent sequencing batches (Supplementary Figure S5). This divergence underscores a critical lesson in transcriptomic discovery: high-variance PCA loadings are not inherently synonymous with stable, reproducible biomarkers.

The reliability of *PDK4*, *HMOX1*, and *TRPV4* as core markers was solidified through a stringent, multi-layer verification protocol designed to filter out stochastic sequencing artifacts. We established that achieving reproducible statistical significance in biomarker detection requires two essential benchmarks: 1) consistency across at least two independent experimental batches and 2) orthogonal validation through RT-PCR. While many high-variance genes were eliminated during cross-batch integration, PDK4 demonstrated remarkable stability, consistently localizing within the significantly upregulated Cluster 2 [C(2)] in the subset of top 1–1,000 transcripts sorted by DEGs (Figure 2) and the Hallmark Xenobiotic Metabolism GSEA pathway (Figure 3). The independent validation of these three markers—confirmed by RT-PCR (Figures 3d,e)—substantiates their role as fundamental regulators of the CH metabolic and inflammatory environment. Specifically, the elevated expression of PDK4 and HMOX1 (Figure 3e) links altered mitochondrial metabolism and oxidative stress responses directly to the underlying disease pathology. By requiring that candidates clear both cross-batch and orthogonal validation tiers, we move beyond “batch-specific” findings toward the identification of truly robust clinical targets. This rigorous workflow ensures that the identified triad of PDK4 and HMOX1 represents a high-confidence transcriptomic signature for chronic hydrocephalus in the elderly caudate nucleus.

Alongside *PDK4* and *HMOX1*, *TRPV4* (LeBlanc et al., 2016; Hochstetler et al., 2020; Toft-Bertelsen et al., 2022) emerged as reproducibly upregulated transcripts in CH, forming a convergent set of RNA markers linked to metabolic stress, oxidative defense, and mechanosensitive signaling. The elevation of these genes aligns with prior reports implicating vascular dysfunction (Ma et al., 2020), mitochondrial stress (Peng et al., 2025), and altered mechanotransduction (Kohler and Hoyer, 2007) in hydrocephalus and related neurovascular disorders (Eide, 2022). Their co-enrichment within xenobiotic metabolism (Kim et al., 2024) and stress-response pathways (Fatmi et al., 2023) further suggests that CH of the elderly is characterized by an adaptive, but potentially maladaptive, metabolic defense state (Su et al., 2026) rather than isolated neurodegeneration (Mallick et al., 2025).

Although the term xenobiotic metabolism can be misleading if interpreted literally, in transcriptomic and neurological contexts its activation does not indicate exposure to pathogens, drugs, or toxins based on diagnosis details provided by the biobank (Supplementary Table S2), but rather reflects endogenous metabolic stress and intrinsic cellular defense responses (Muralidharan and Mandrekar, 2013). Gene set enrichment analyses reinforce this interpretation by identifying xenobiotic metabolism as a dominant disease-associated pathway, with *PDK4* and *HMOX1* as leading-edge contributors (Perea-Martinez et al., 2022). In neurological contexts, activation of xenobiotic metabolism is increasingly recognized as a surrogate for endogenous metabolic stress, mitochondrial dysfunction, and oxidative challenge (Durairaj and Liu, 2025). The persistence of xenobiotic, immune, inflammatory, and structural remodeling pathways across rank-stratified gene windows extending well beyond the top 1,000 genes indicates that these programs are deeply embedded within the CH transcriptome. This broad distribution argues against stochastic expression noise and instead supports a model of sustained, systems-level transcriptional adaptation.

From a translational perspective, these findings have important implications for fluid-based biomarker development (Pyykko et al., 2014). While the present study focused on brain tissue, the prominence of *PDK4*—a metabolic regulator with established expression in peripheral tissues—raises the possibility that *PDK4* or *PDK4*-driven transcriptional signatures in blood or CSF may serve as informative biomarkers of CH. Unlike conventional protein markers that primarily reflect downstream neuronal injury, *PDK4*-associated pathways may capture upstream metabolic dysregulation, offering a mechanistically grounded and potentially earlier indicator of disease activity. This is particularly relevant for CH, where diagnosis and monitoring remain challenging and largely reliant on imaging and clinical response to CSF diversion (Adil et al., 2024; De la Cerda-Vargas et al., 2024; Kadaba Sridhar et al., 2024; Alotaibi and Fasano, 2025; Chen et al., 2025; Emery et al., 2025; Gholampour et al., 2025; Harbaugh et al., 2025; Liu et al., 2025; Menberu et al., 2025; Nishizawa et al., 2025; Rosu et al., 2025; Sachithanandan et al., 2025; Safdar et al., 2025; Yamada, 2025).

Our evolutionary genomic analyses further contextualize the prominence of *PDK4* by demonstrating that it resides within genomic architectures permissive to regulatory variability (McKnight et al., 2021; McKnight et al., 2023; Cunningham et al., 2025). Across mouse, rat, pig, and human genomes, *PDK4* exhibits progressive enrichment toward telomeric regions and increasing A+T nucleotide composition, reaching >59% A+T content in humans (Nusbaum et al., 2006), a feature associated with elevated mutability and transcriptional plasticity. These properties suggest that PDK4 may be especially responsive to pathological stress in the aging human brain. Importantly, this finding also highlights a potential limitation of current animal models: while rodents and pigs are widely used to model adult hydrocephalus, their lower A+T content and differing chromosomal contexts may constrain the extent to which *PDK4* regulation fully recapitulates human disease biology.

The findings presented in this work reveal that hydrocephalus (CH) in the elderly is associated with pervasive and hierarchical transcriptomic reprogramming rather than isolated gene-level perturbations. The convergence of metabolic stress, immune activation, and structural remodeling across multiple rank-stratified gene sets supports a model of sustained, systems-level alteration of caudate nucleus biology. Notably, the emergence of PDK4-centered metabolic signatures within evolutionarily permissive genomic contexts suggests a mechanistic link between energy metabolism, hypoxia, and neurovascular dysfunction. Given the roles of VEGF and HB-EGF in maintaining vascular integrity for human brains (Chu et al., 2011; Shim et al., 2013b; Shim et al., 2014; Huang et al., 2016; Shim et al., 2016; Shen et al., 2022), these observations provide a framework for interpreting CH not as a focal process, but as a chronic, multifactorial brain stress state where metabolic–immune crosstalk is mediated by the physiological demands of the aging cerebral vasculature.

A central motivation of this study was to identify disease-relevant transcriptional programs that could serve as a foundation for minimally invasive RNA biomarkers. Using postmortem caudate nucleus tissue, we demonstrate that CH involves robust reprogramming of pathways—particularly xenobiotic metabolism and cytokine signaling—that reflect systemic and neurovascular processes. This makes them especially well-suited for translation into blood- or CSF-based RNA assays. From a clinical perspective, detecting these signatures would represent a significant advance in earlier diagnosis and objective monitoring of treatment response following shunt placement. Moreover, fluid-based RNA biomarkers could provide the molecular resolution needed to distinguish CH from irreversible neurodegenerative conditions like Alzheimer’s or Parkinson’s disease, addressing a critical unmet need in the differential diagnosis of cognitive impairment.

Several limitations warrant consideration. While bulk RNA-seq provides a comprehensive systems overview, it precludes cell-type-specific resolution. Future studies integrating single-nucleus transcriptomics or spatial profiling will be essential to localize PDK4 and related pathways to specific neuronal, glial, or vascular compartments—particularly the endothelial-mural cell interface. Additionally, while our cohort size is appropriate for discovery, longitudinal studies are required to relate PDK4 expression to disease duration and clinical outcomes. Our next steps will focus on validating these brain-derived candidates in matched blood and CSF samples using platforms optimized for low-abundance transcripts (e.g., 1–10 FPKM). Integrating molecular data with neuroimaging metrics will be essential to establish a prognostic utility that honors the complex hemodynamics of the hydrocephalic brain.

In summary, this study demonstrates that CH in the elderly is characterized by robust, multilayered transcriptomic reprogramming, with PDK4 emerging as a central marker embedded within metabolic stress pathways. The convergence of transcriptomic discovery and evolutionary genomic analyses highlights PDK4 as both a mechanistic indicator of disease biology and a promising translational target. Together, these findings provide a biologically grounded framework for understanding CH as a sustained metabolic–vascular–immune stress state, laying the groundwork for future RNA-based biomarkers and stratified therapeutic approaches.

## Methods

### Human postmortem brain tissue

Human postmortem brain tissues were obtained from the National Institutes of Health (NIH) NeuroBioBank (NBB), USA, over a 1-year acquisition period (Supplementary Table S1). Frozen caudate nucleus specimens were collected from multiple NBB repositories and shipped to the laboratory under continuous frozen conditions. According to NBB documentation, postmortem intervals were 16 ± 8 h (mean ± SD; range: 4–25 h). The study cohort consisted of n = 12 donors, including n = 6 neurologically unaffected aged controls and n = 6 individuals with chronic hydrocephalus (CH) (Supplementary Table S2). The cohort included six male and six female donors, with ages ranging from 72 to 88 years. Most donors were White (n = 11), with one African American donor in the CH group. Clinical diagnoses and relevant medical histories were obtained from NBB records and are summarized in Supplementary Table S2. Control cases had no documented neurological disease, although one donor had a non-neurological medical history (gall bladder disease). Among CH cases, documented etiologies or comorbidities included idiopathic hydrocephalus, traumatic brain injury, multiple sclerosis, cerebral atherosclerosis, and vascular dementia, as indicated in Supplementary Table S2. All tissues were fully de-identified prior to receipt and were handled in accordance with NIH NeuroBioBank policies and institutional ethical guidelines.

### Whole-transcriptome RNA sequencing outlines

Bulk whole-transcriptome RNA sequencing (RNA-seq) was performed in two independent sequencing sessions to minimize batch effects and assess reproducibility, interrogating a total of roughly 40,000 annotated genetic loci. In the first sequencing session (N = 4; n = 2 control, n = 2 CH), roughly 3% met nominal statistical significance (P < 0.05), with *PDK4* among the most significant. In the second session (N = 6; n = 3 per group), approximately 10% genes were significant at P < 0.05, and 4% met a more stringent threshold (P < 0.01). Across both sessions, genes involved in xenobiotic metabolism, adipogenesis, and hypoxia pathway signaling molecules ranked top 3 highly distinguishable sets based on statistical significance and effect size. Genes were ranked by variance and differential expression metrics for downstream principal component analysis (PCA), hierarchical clustering, and rank-stratified gene set enrichment analyses.

### Bioinformatics analysis of RNA sequencing reads (DESeq2)

Raw reads were trimmed to remove adapter content and low-quality base calls using Trimmomatic version 0.39 (Bolger et al., 2014). Resulting trimmed reads were aligned to the reference human genome GRCh38 using HISAT2 version 2.2.1 (Kim et al., 2019). Read counts per gene per sample were generated using the R/Bioconductor package GenomicAlignments version 1.30.0 and differentially expressed genes were identified using DESeq2 version 1.34.0 (Love et al., 2014) with a false discovery rate threshold of 0.1. Results were exported from R to comma separated value format for downstream processing. Pathway analysis was performed by processing the full result set from DESeq2 with the Gene Set Enrichment (GSEA) algorithm (Subramanian et al., 2005) as implemented by the R/Bioconductor package fgsea version 1.20.0, against the Reactome pathways database (Ragueneau et al., 2026).

### Primer design

Primers were designed for six target genes and one housekeeping gene using exon-specific transcript information from the Ensembl database (https://useast.ensembl.org) and Primer3 software (https://bioinfo.ut.ee/primer3-0.4.0/). Exon selection prioritized transcript regions conserved across splice variants where applicable. Lyophilized primers were synthesized by Thermo Fisher Scientific (Waltham, MA). Primer sequences, amplicon sizes, and genomic coordinates are listed in Supplementary Table S3).

### RNA isolation and cDNA synthesis

Total RNA was isolated from frozen caudate nucleus tissue using the RNeasy Lipid Tissue Mini Kit (QIAGEN) with a QIAzol-based protocol, optimized for lipid-rich brain tissue. RNA concentration and purity were assessed using a NanoDrop spectrophotometer (Thermo Fisher Scientific). For cDNA synthesis, 500 ng of total RNA per sample was reverse-transcribed using the High-Capacity RNA-to-cDNA Kit (Thermo Fisher Scientific; Cat. No. 4368814) on an ABI SimpliAmp Thermal Cycler, following the manufacturer’s protocol and the previous reports (Shim et al., 2013a; White et al., 2022; Barrett et al., 2024; Cunningham et al., 2025).

### Reverse transcription polymerase chain reaction (RT-PCR)

RT-PCR was conducted in 25 μL reaction volumes containing 250 ng of cDNA using GoTaq® Green Master Mix (Promega, Madison, WI). Thermal cycling conditions consisted of an initial denaturation at 95 °C for 2 min, followed by 35 cycles of 95 °C for 30 s, 60 °C for 30 s, and 72 °C for 30 s. PCR products were resolved on 1.25% agarose gels in 1× TBE buffer and visualized using MaestroSafe dye (Maestrogen) on a FluorChem E imaging system (Bio-Techne).

### Image quantification

Gel images were quantified using NIH ImageJ software. Analysis steps included lane selection, densitometric profile plotting, background subtraction, peak integration, and calculation of band intensities. Target gene expression was normalized to the housekeeping gene, and normalized values were exported for statistical analysis following the previous methods (Barrett et al., 2024; Cunningham et al., 2025).

### Statistical analysis

Statistical analyses and PCA were performed using GraphPad Prism (version 10.6.1). Given the modest sample size and non-normal data distribution, non-parametric tests were used throughout. Two-group comparisons employed the Mann–Whitney U test with *post hoc* testing as appropriate. Statistical significance was defined as P < 0.05, with significance levels denoted as *P < 0.05, **P < 0.01, and ***P < 0.005.

### Gene set enrichment, hierarchical clustering, and rank-stratified analysis

Gene set enrichment analysis (GSEA) was conducted using GSEA software (v4.3.3) with Hallmark gene sets from MSigDB, supplemented by analyses using g:Profiler. Enrichment significance was assessed using permutation-based normalized enrichment scores. Hierarchical clustering and module detection were performed using Instant Clue software, generating dendrograms and heatmaps to visualize co-expression patterns across disease groups (Barrett et al., 2024; Cunningham et al., 2025). To evaluate the persistence of biological pathways across transcriptional layers, genes were analyzed in rank-stratified windows (e.g., top 1–1,000; 1,001–2,000; subsequent intervals), as described in Supplementary Figures S1–S20.

### Genomic mutability and comparative genome architecture analysis

To assess whether key disease-associated genes exhibit intrinsic genomic features that may predispose them to dysregulation, genomic mutability analyses were performed following analytical frameworks previously established and validated by our group (Lucas et al., 2021; McKnight et al., 2021; Raines et al., 2022; White et al., 2022; Hart et al., 2023; McKnight et al., 2023; Barrett et al., 2024; Cunningham et al., 2025). For selected candidate genes (including PDK4 and comparator loci), transcript length, chromosomal location, proximity to telomeric regions, and nucleotide composition (A+T content) were extracted from publicly available genome annotations for mouse, rat, pig, and human. Chromosome length distributions and relative karyotype architectures were analyzed to contextualize gene localization within species-specific genomic landscapes. These features were evaluated descriptively and comparatively to infer relative regulatory plasticity and mutational susceptibility across species, rather than to detect somatic mutations *per se*. This approach enabled integration of transcriptomic dysregulation with evolutionary and genomic context. Together, this integrated methodological pipeline—combining bulk RNA-seq, rank-stratified enrichment analysis, RT-PCR validation, and comparative genomic mutability assessment—enabled systematic identification of disease-associated transcriptional programs in chronic hydrocephalus and established a foundation for translational extension into blood- and CSF-based RNA biomarker assays.

## Data Availability

The original contributions presented in the study are included in the article/Supplementary Material. Structured datasets supporting the findings of this study are available in the Supplementary Material. The primary RNA-seq dataset comparing “Control and CH specimens” is hosted at Harvard Dataverse (https://dataverse.harvard.edu/dataset.xhtml?persistentId=doi:10.7910/DVN/VUJR5O). Please note that PDK4-specific transcriptomic data has been excluded from the public repository in accordance with a pending Invention Disclosure (https://data.uspto.gov/patent-file-wrapper/search/details/PCTUS2532689/application-data?fbclid=IwY2xjawP_VldleHRuA2FlbQIxMABicmlkETFSZ0lTOVE4bWRsUzZVWDNYc3J0YwZhcHBfaWQQMjIyMDM5MTc4ODIwMDg5MgABHlgkXWB6Hu_dArs-FKmwc33jTsCJZhOVhlHRg0_rJ6NymQ-kA2JRRh3aoUUu_aem_WJWtGO). Raw or processed RNA-seq data for PDK4 are available from the corresponding author (JS) upon reasonable request and subject to a standard data-use agreement.
