# Supplementary material for: PDK4 as a metabolic biomarker of chronic hydrocephalus
Source: Front Genet. 2026 Mar 19;17:1780231. doi: 10.3389/fgene.2026.1780231 (PMC13043410; doi:10.3389/fgene.2026.1780231)
Supplement: Supplementary file 1 [file DataSheet1.pdf]

Supplementary Material for

PDK4 as a Metabolic Biomarker of Chronic Hydrocephalus

Robbie Clarke[1], Payton Villers[1], Chloe Bills[1], Michaela Rice[1], Madison Higgins[1], Chan Lee[2], Prabir Patra[1], Peter H-U Lee[3,4], Abhay Moghekar[5], Joon W. Shim[1*]

Correspondence to: shim@marshall.edu

This file includes:

Figure S1 to S20;
Tables S1 to S8

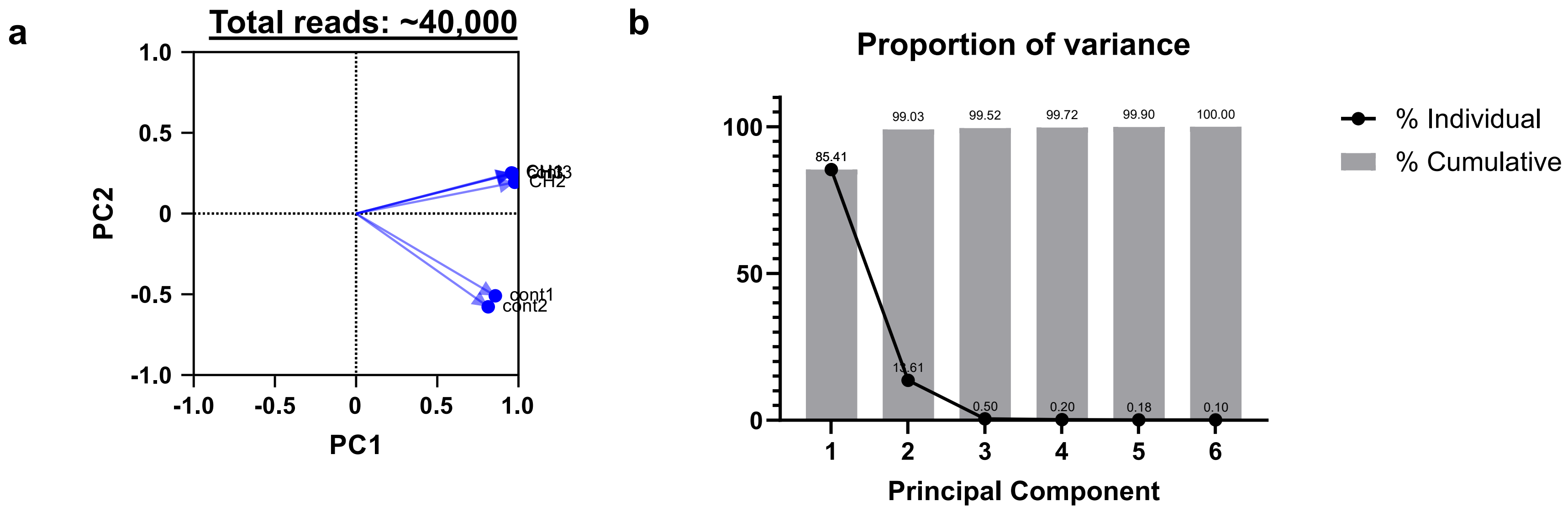

Figure S1. Principal Component Analysis (PCA) of Global Gene Expression Profiles.
This figure illustrates the variance and clustering of gene expression data across different experimental groups for the total dataset (~40,000 reads).
(a) PCA Loadings Plot: Displays the relationship between the original variables (samples) and the first two principal components (PC1 and PC2). The vectors for experimental groups (e.g., CH1, CH2, CH3 vs. cont1, cont2) indicate their contribution to the principal components and their directional correlation. Samples from the chronic hydrocephalus (CH) group cluster in the upper right quadrant, while control samples (cont1, cont2) are localized in the lower right, indicating distinct transcriptional programs.
(b) Scree Plot of Proportion of Variance: Quantifies the percentage of total variance explained by each principal component to validate the use of 2D projections.
Individual Variance (line graph): Shows that PC1 accounts for the vast majority of the variance (85.41%), while PC2 accounts for 13.61%.
Cumulative Variance (bar graph): Demonstrates that the first two components combined explain over 99% of the total variance in the dataset, suggesting that the two-dimensional plot in panel (a) is highly representative of the overall data structure.

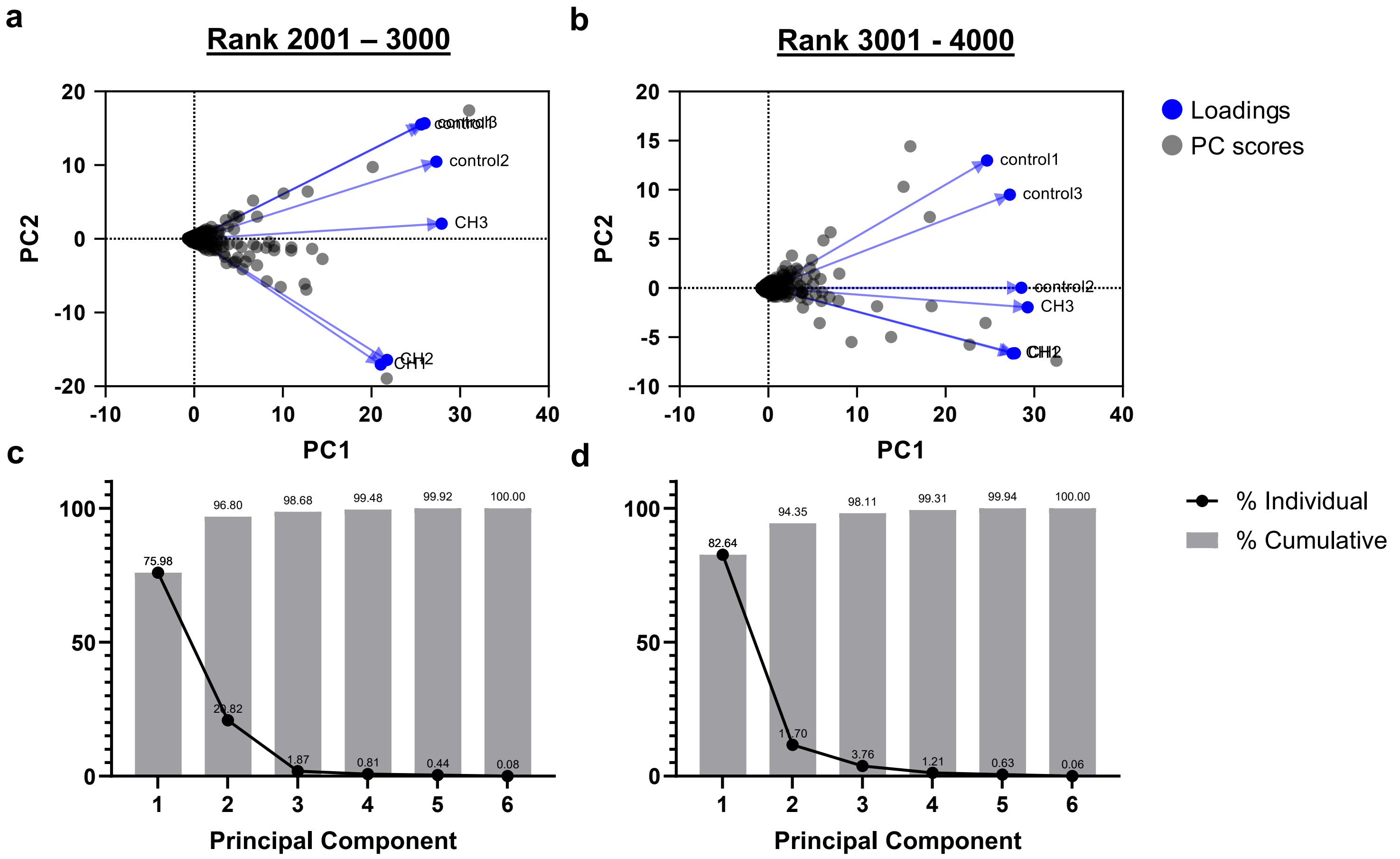

Figure S2. Comparative Analysis of Mid-Range Gene Sets (Rank 2001 – 3000 & 3001 – 4000). (a) Biplot (Rank 2001 – 3000): Shows the relationship between mid-ranked genes and experimental groups. Vectors indicate a clear separation between control and CH samples along both axes. (b) Biplot (Rank 3001 – 4000): Illustrates the distribution of genes in the 3001–4000 rank range, maintaining similar directional separation between control and experimental groups. (c) Scree Plot (Rank 2001 – 3000): PC1 accounts for 75.98% of the variance, with a cumulative 96.80% explained by the first two components. (d) Scree Plot (Rank 3001 – 4000): PC1 accounts for 82.64% of the variance, with a cumulative 94.35% explained by the first two components.

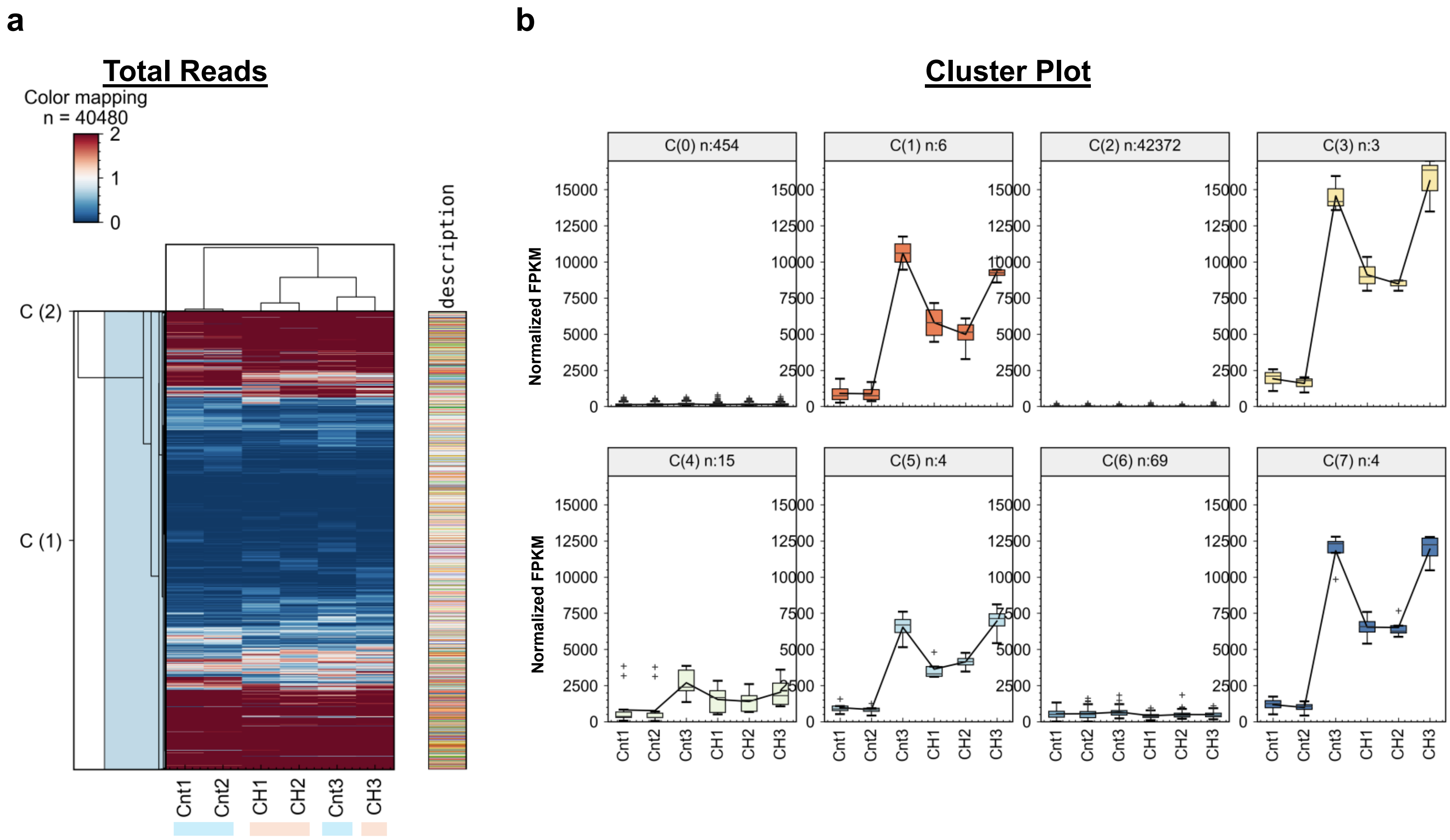

Figure S3. Global Transcriptomic Profile of Total RNA-Seq Reads.
** (a) Heatmap of Total Reads:** Hierarchical clustering of all transcripts (n = 40,480) reveals primary gene clusters **C(1)** and **C(2)**. At this global scale, inter-sample heterogeneity prevents strict binary diagnostic segregation (Control vs. CH) in the column dendrogram. (b) Segmentation into eight expression trajectories based on **Normalized FPKM**. Most transcripts reside in the stable **C(2)** cluster (n = 42,372). High-variance clusters **C(1)**, **C(3)**, and **C(7)** identify high-magnitude drivers (exceeding 15,000 FPKM) that anchor the pathological signature of chronic hydrocephalus.

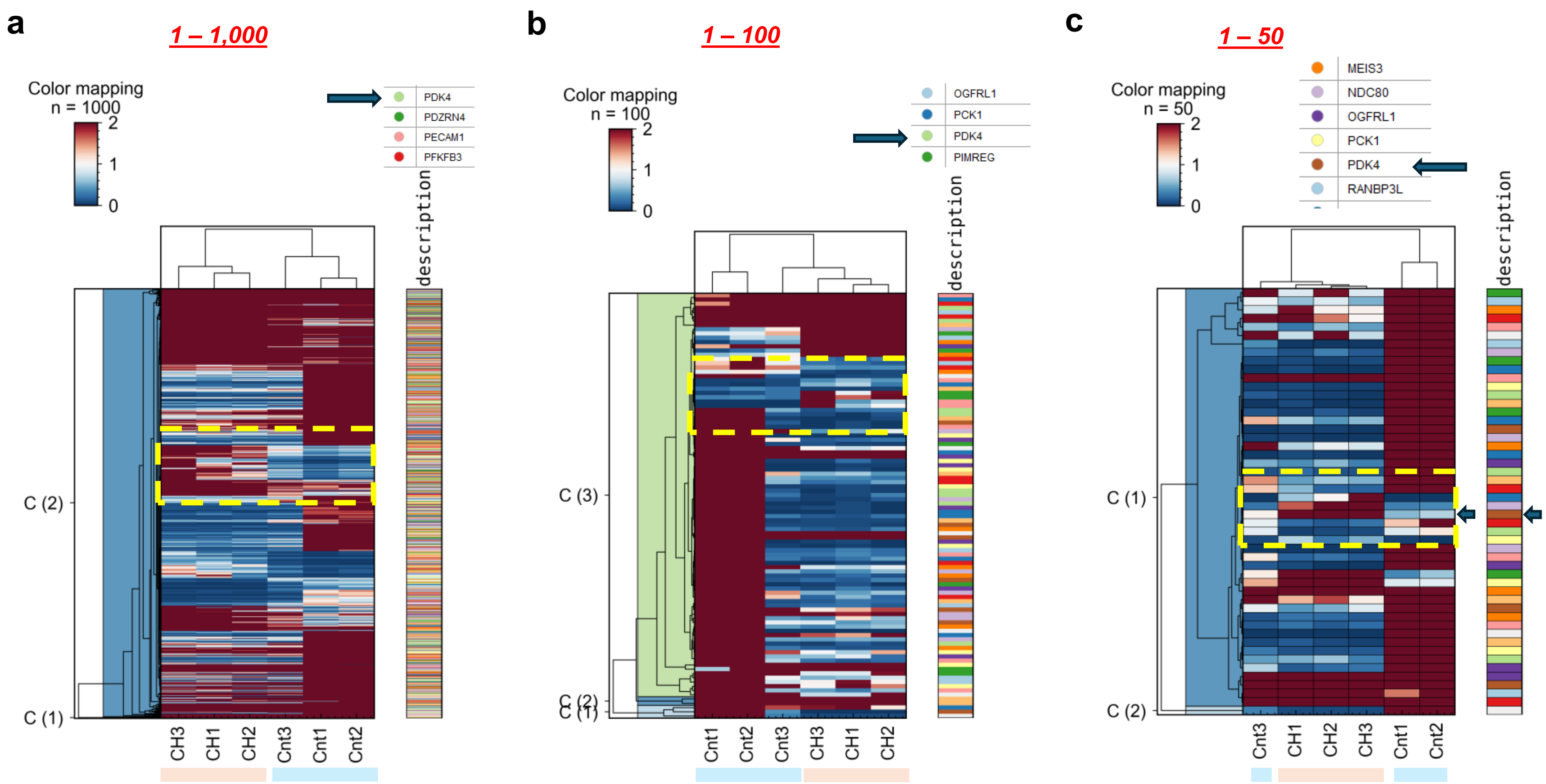

Figure S4. Comparative Hierarchical Clustering Across Varying Gene Scopes. The figure illustrates the multi-scale re-analysis of differentially expressed genes (DEGs), highlighting the relationship between data magnification and diagnostic segregation.

(a) Top 1,000 DEGs: Global hierarchical clustering demonstrates a primary split into clusters C(1) and C(2). At this scale, PDK4 (indicated by arrow) is embedded within a high-density cluster, representing a broad transcriptomic signature.

(b) Top 100 DEGs: Refined clustering shows a realignment into three major branches [C(1)–C(3)]. Despite the narrowed scope, PDK4 remains a stable component of the pathological signature, identified by its distinct color pattern relative to the control group.

(c) Top 50 DEGs: At this high-resolution level, automated hierarchical clustering fails to maintain strict binary segregation between control (Cnt) and chronic hydrocephalus (CH) groups in the column dendrogram. However, the specific location of PDK4 (yellow box) remains clearly discernible through its unique color mapping pattern, serving as a consistent landmark across all levels of analysis.

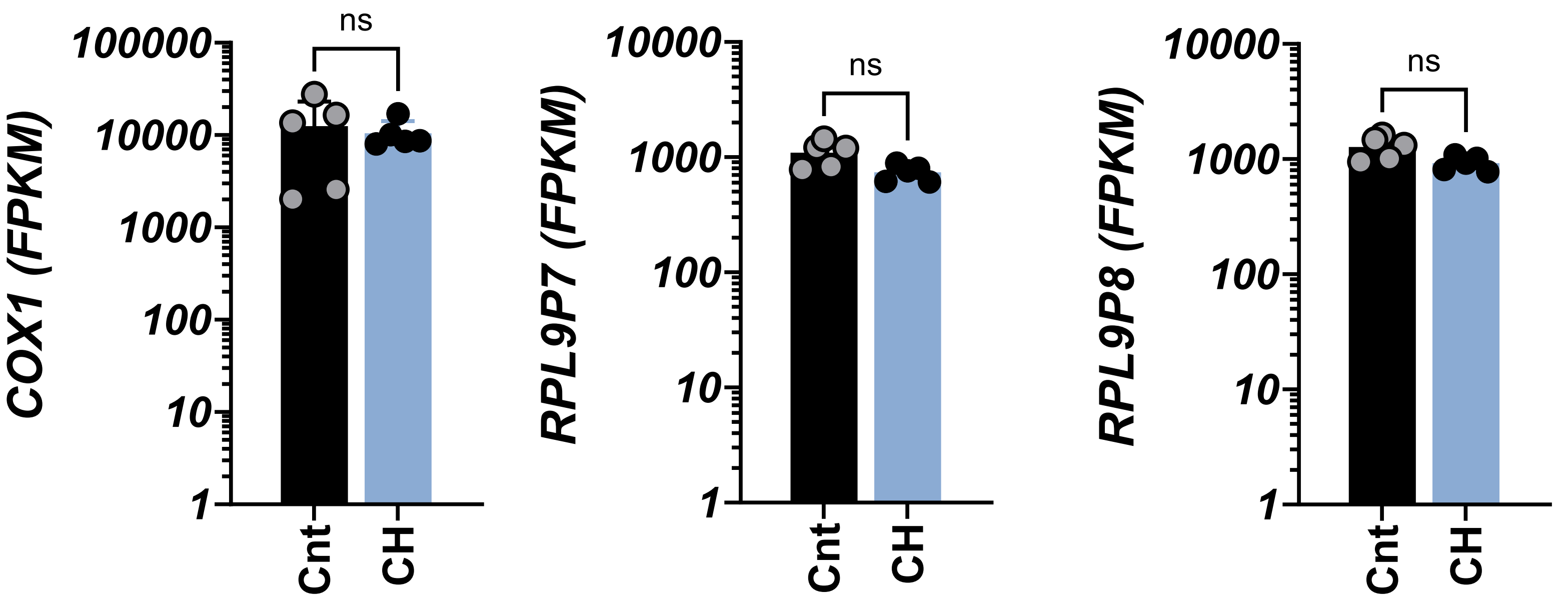

Figure S5. RNA-seq results of two batches on three genes (batch one: n=3/group; batch two: n=2/group). Normalized expression levels (FPKM) for the primary PC1 loadings found in Fig. 1: ***COX1***, ***RPL9P7***, and ***RPL9P8***. Despite their heavy influence on PCA clustering, individual comparisons show no statistically significant (ns) differences in mean expression between Cnt and CH groups when the two batches are integrated.

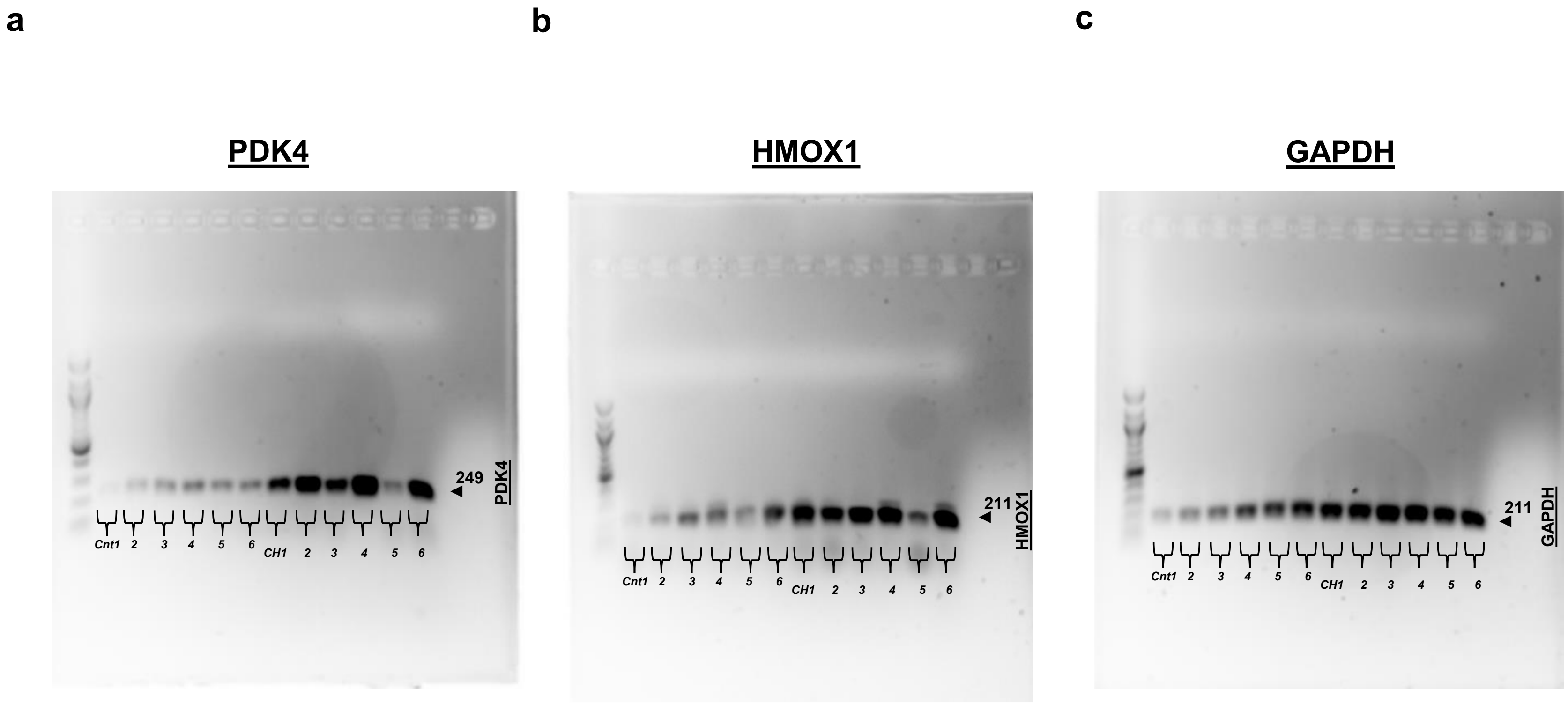

Figure S6. Whole gel images of semi-quantitative RT-PCR validation: PDK4 and HMOX1 expression in the caudate nucleus of chronic hydrocephalus. Representative agarose gel electrophoresis images showing RT-PCR amplification of **PDK4** (panel a), **HMOX1** (panel b), and the housekeeping control **GAPDH** (panel c) from postmortem human caudate nucleus tissue. Samples include age-matched neurologically normal controls (**Cnt1–Cnt6**) and individuals with chronic hydrocephalus (**CH1–CH6**). Consistent with bulk RNA-sequencing results, **PDK4 and HMOX1 transcripts exhibit increased band intensity in CH samples relative to controls**, indicating upregulation of metabolic and oxidative stress–responsive genes in chronic hydrocephalus. GAPDH expression remains stable across samples, confirming comparable RNA input and cDNA synthesis efficiency. Arrowheads indicate the expected amplicon sizes (PDK4: 249 bp; HMOX1: 211 bp; GAPDH: 211 bp).

a b c

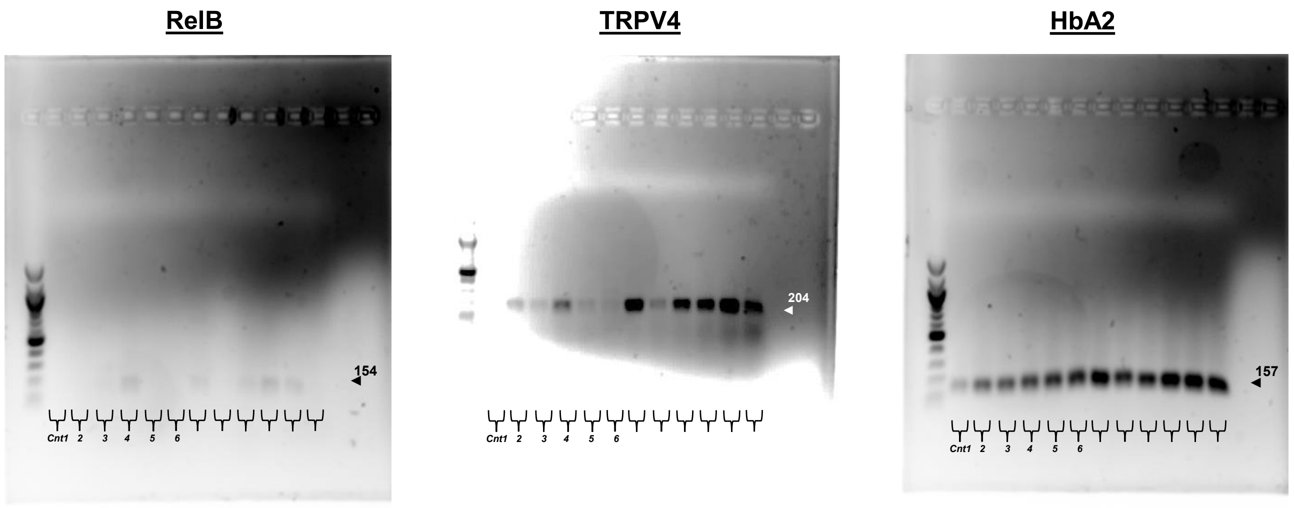

b’

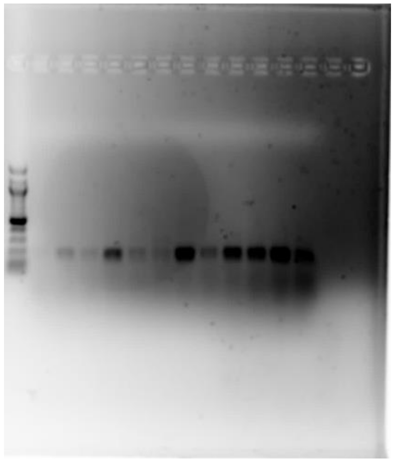

Figure S7. Semi-quantitative RT-PCR validation of candidate transcripts in the caudate nucleus.
Representative agarose gel electrophoresis images showing RT-PCR amplification of three candidate genes identified from bulk RNA-sequencing analysis of postmortem human caudate nucleus tissue. Lanes include age-matched neurologically normal controls and chronic hydrocephalus (CH) samples, loaded in identical order across all panels. Arrowheads denote the expected amplicon sizes for each target transcript (154 bp, 204 bp, and 157 bp, respectively). Panel b′ shows the original, unprocessed gel images acquired under identical exposure conditions for qualitative comparison. Panel b displays the corresponding images after contrast enhancement using PowerPoint image adjustment tools, which were subsequently used for semi-quantitative densitometric analysis in NIH ImageJ to improve band delineation and measurement accuracy. Band intensity patterns demonstrate reproducible amplification and differential expression trends consistent with RNA-seq–based transcript ranking, supporting the validity of candidate gene selection. Molecular weight markers are shown at the left of each gel.

Rank 2

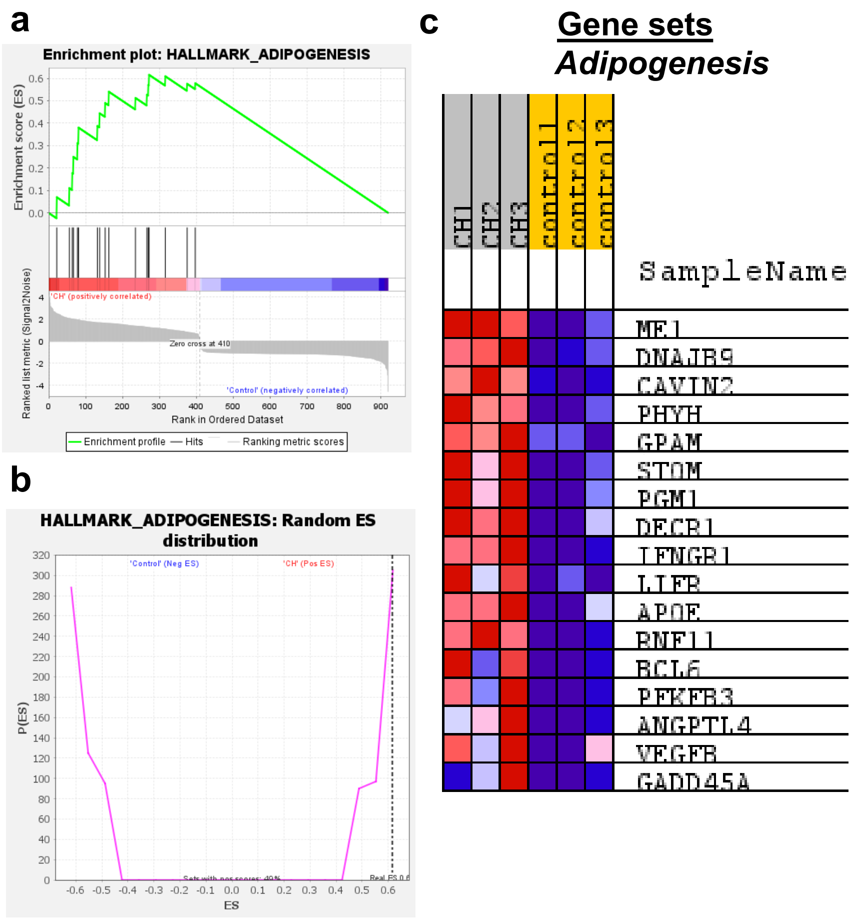

Figure S8. Enrichment of adipogenesis-related transcriptional programs in chronic hydrocephalus.
Gene Set Enrichment Analysis (GSEA) of bulk RNA-sequencing data from postmortem human caudate nucleus reveals significant positive enrichment of the **HALLMARK_ADIPOGENESIS** pathway in chronic hydrocephalus (CH) relative to age-matched neurologically normal controls. **Top left**, enrichment score (ES) plot showing adipogenesis-associated genes concentrated toward the CH-correlated end of the ranked gene list. Vertical black bars indicate the positions of individual gene set members within the ordered dataset. **Bottom left**, random enrichment score distribution demonstrating that the observed ES lies outside the null distribution, confirming statistical significance. **Right**, heatmap of leading-edge adipogenesis genes driving the enrichment signal, including metabolic, lipid-handling, and stress-responsive transcripts (e.g., *GPAM, PGM1, APOE, VEGFB,* and *ANGPTL4*). Expression values are Z-score–normalized across samples. CH samples cluster distinctly from controls, indicating coordinated upregulation of adipogenic and lipid-metabolic gene programs associated with chronic hydrocephalus pathology.

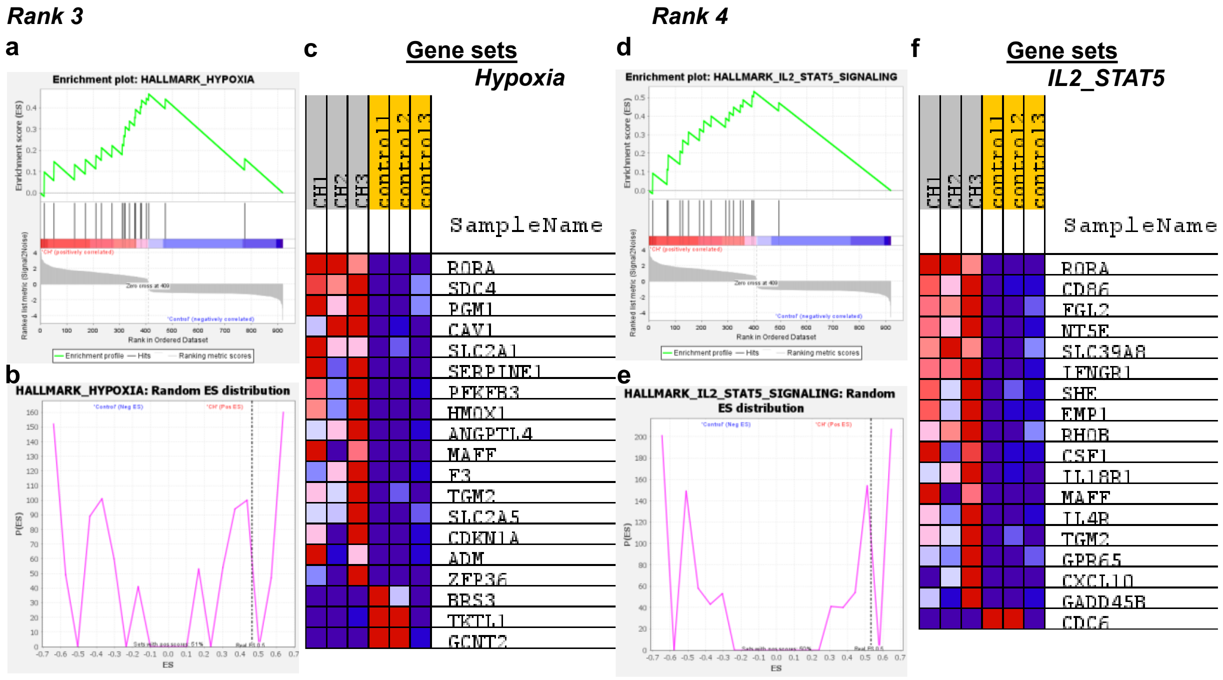

Figure S9. Gene Set Enrichment Analysis (GSEA) of hypoxia and IL2–STAT5 signaling pathways.

(a) GSEA enrichment plot for the HALLMARK_HYPOXIA gene set showing positive enrichment in the CH group relative to controls, based on ranked differential expression of the top 1,000 genes.

(b) Permutation-based random enrichment score (ES) distribution for the HALLMARK_HYPOXIA gene set, with the observed ES indicated by a dashed vertical line.

(c) Heatmap of leading-edge genes contributing to hypoxia signaling, displaying relative expression levels across CH and control samples.

(d) GSEA enrichment plot for the HALLMARK_IL2_STAT5_SIGNALING gene set, demonstrating positive enrichment in the CH group.

(e) Random ES distribution for the HALLMARK_IL2_STAT5_SIGNALING gene set derived from permutation testing.

(f) Heatmap of leading-edge genes associated with IL2–STAT5 signaling, illustrating coordinated upregulation in CH samples compared with controls.

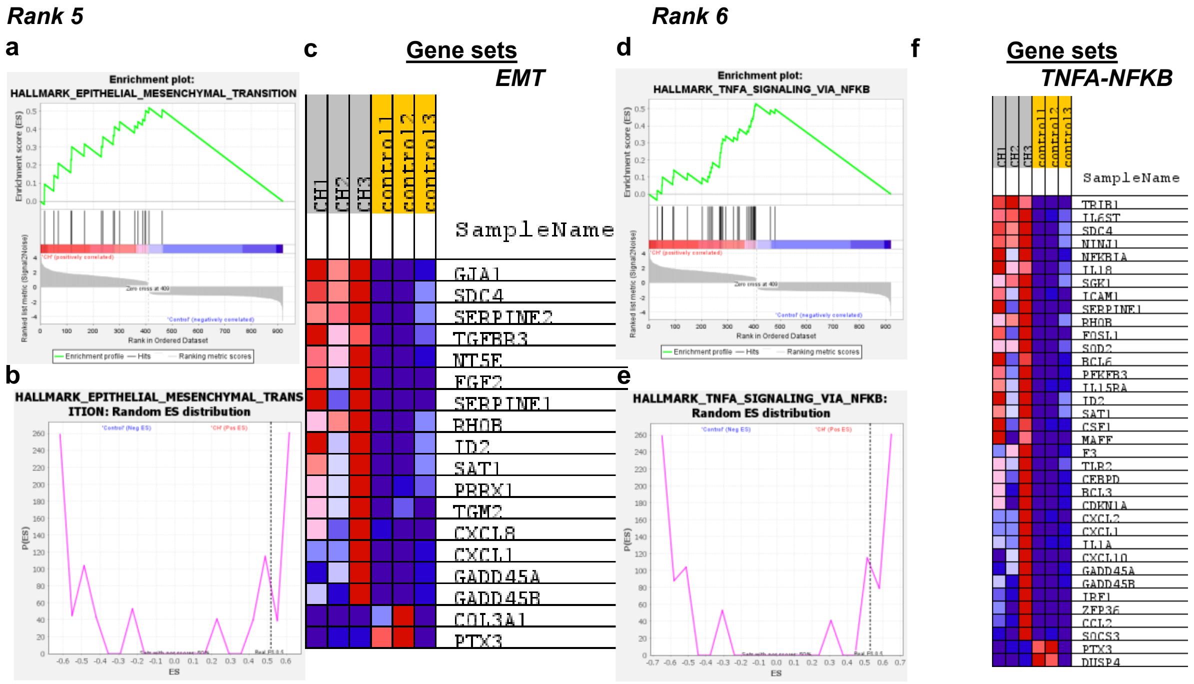

Figure S10. Gene Set Enrichment Analysis (GSEA) of epithelial–mesenchymal transition (EMT) and TNFα–NFκB signaling pathways.

(a) GSEA enrichment plot for the HALLMARK_EPITHELIAL MESENCHYMAL TRANSITION gene set, demonstrating positive enrichment in the CH group relative to controls based on ranked differential expression of the top 1,000 genes.

(b) Permutation-based random enrichment score (ES) distribution for the EMT gene set, with the observed ES indicated by a dashed vertical line.

(c) Heatmap of leading-edge genes contributing to EMT signaling, showing coordinated upregulation in CH samples compared with controls.

(d) GSEA enrichment plot for the HALLMARK_TNFA_SIGNALING_VIA_NFKB gene set, indicating significant enrichment in the CH group.

(e) Random ES distribution for the TNFα–NFκB signaling gene set derived from permutation testing.

(f) Heatmap of leading-edge genes associated with TNFα–NFκB signaling, highlighting inflammatory and stress-responsive transcriptional programs activated in CH samples.

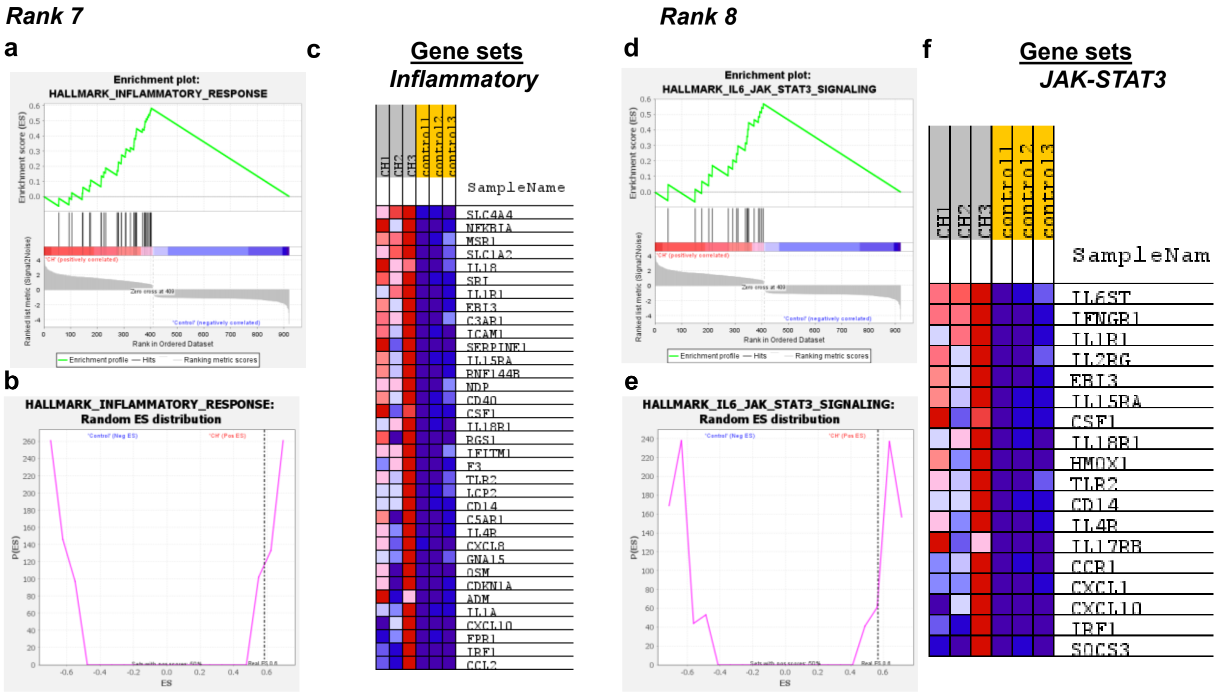

Figure S11. Gene Set Enrichment Analysis (GSEA) of inflammatory response and IL-6–JAK–STAT3 signaling pathways.

(a) GSEA enrichment plot for the HALLMARK_INFLAMMATORY_RESPONSE gene set, showing positive enrichment in the CH group relative to controls based on ranked differential expression of the top 1,000 genes.

(b) Permutation-based random enrichment score (ES) distribution for the inflammatory response gene set, with the observed ES indicated by a dashed vertical line.

(c) Heatmap of leading-edge genes contributing to the inflammatory response, demonstrating coordinated upregulation of immune and cytokine-related genes in CH samples compared with controls.

(d) GSEA enrichment plot for the HALLMARK_IL6_JAK_STAT3_SIGNALING gene set, indicating significant enrichment in the CH group.

(e) Random ES distribution for the IL-6–JAK–STAT3 signaling gene set derived from permutation testing.

(f) Heatmap of leading-edge genes associated with IL-6–JAK–STAT3 signaling, highlighting activation of cytokine-driven and STAT3-dependent transcriptional programs in CH samples.

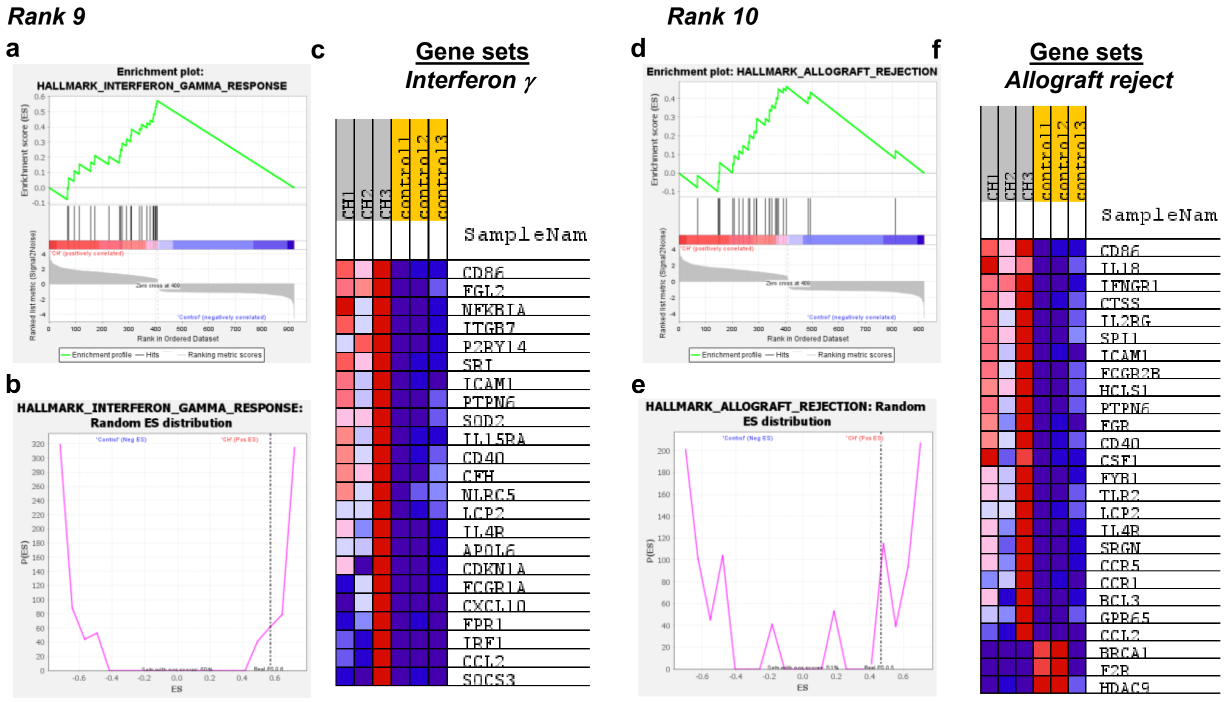

Figure S12. Gene Set Enrichment Analysis (GSEA) of interferon-γ response and allograft rejection pathways.

(a) GSEA enrichment plot for the HALLMARK_INTERFERON_GAMMA_RESPONSE gene set, showing positive enrichment in the CH group relative to controls based on ranked differential expression of the top 1,000 genes.

(b) Permutation-based random enrichment score (ES) distribution for the interferon-γ response gene set, with the observed ES indicated by a dashed vertical line.

(c) Heatmap of leading-edge genes contributing to interferon-γ signaling, demonstrating coordinated upregulation of immune activation and antigen-presentation–related genes in CH samples compared with controls.

(d) GSEA enrichment plot for the HALLMARK_ALLOGRAFT_REJECTION gene set, indicating significant enrichment in the CH group.

(e) Random ES distribution for the allograft rejection gene set derived from permutation testing.

(f) Heatmap of leading-edge genes associated with the allograft rejection pathway, highlighting activation of adaptive and innate immune response programs in CH samples.

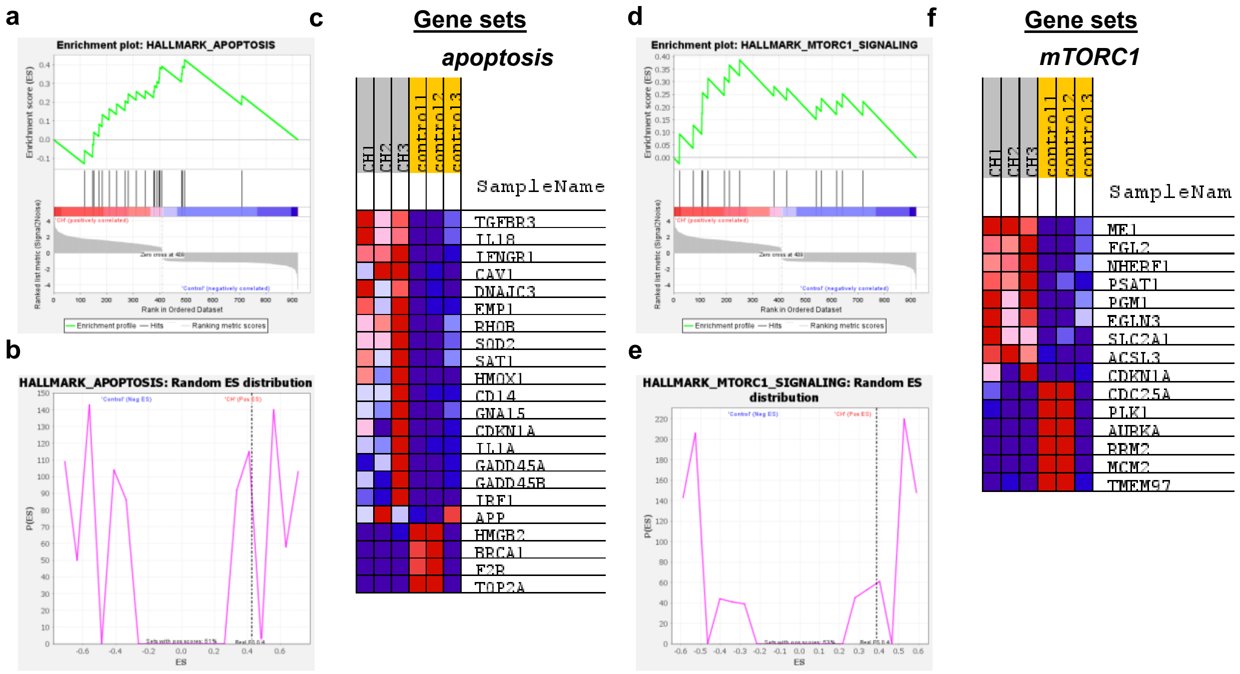

Figure S13. Gene Set Enrichment Analysis (GSEA) of apoptosis and mTORC1 signaling pathways.

(a) GSEA enrichment plot for the **HALLMARK_APOPTOSIS** gene set, showing positive enrichment in the CH group relative to controls based on ranked differential expression of the top 1,000 genes.

(b) **Permutation-based random enrichment score (ES) distribution** for the apoptosis gene set, with the observed ES indicated by a dashed vertical line.

(c) **Heatmap of leading-edge genes** contributing to apoptotic signaling, demonstrating coordinated regulation of cell death– and stress-related genes in CH samples compared with controls.

(d) GSEA enrichment plot for the **HALLMARK_MTORC1_SIGNALING** gene set, indicating significant enrichment in the CH group.

(e) **Random ES distribution** for the mTORC1 signaling gene set derived from permutation testing.

(f) **Heatmap of leading-edge genes** associated with mTORC1 signaling, highlighting altered metabolic, proliferative, and growth-related transcriptional programs in CH samples.

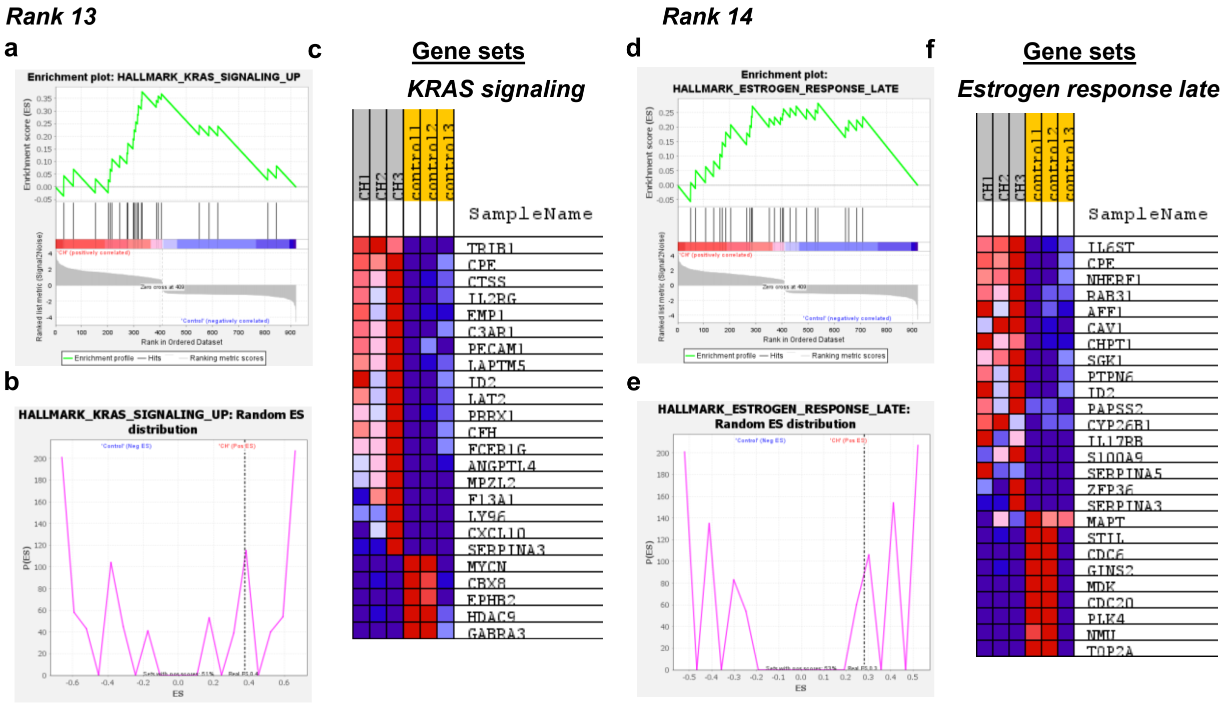

Figure S14. Gene Set Enrichment Analysis (GSEA) of KRAS signaling and estrogen response pathways.

(a) GSEA enrichment plot for the **HALLMARK_KRAS_SIGNALING_UP** gene set, demonstrating positive enrichment in the CH group relative to controls based on ranked differential expression of the top 1,000 genes.

(b) **Permutation-based random enrichment score (ES) distribution** for the KRAS signaling gene set, with the observed ES indicated by a dashed vertical line.

(c) **Heatmap of leading-edge genes** contributing to KRAS signaling, showing coordinated regulation of growth, stress-response, and signal transduction–related genes in CH samples compared with controls.

(d) GSEA enrichment plot for the **HALLMARK_ESTROGEN_RESPONSE_LATE** gene set, indicating significant enrichment in the CH group.

(e) **Random ES distribution** for the estrogen response gene set derived from permutation testing.

(f) **Heatmap of leading-edge genes** associated with late estrogen response, highlighting altered transcriptional programs involved in hormonal signaling, metabolism, and cell-cycle regulation in CH samples.

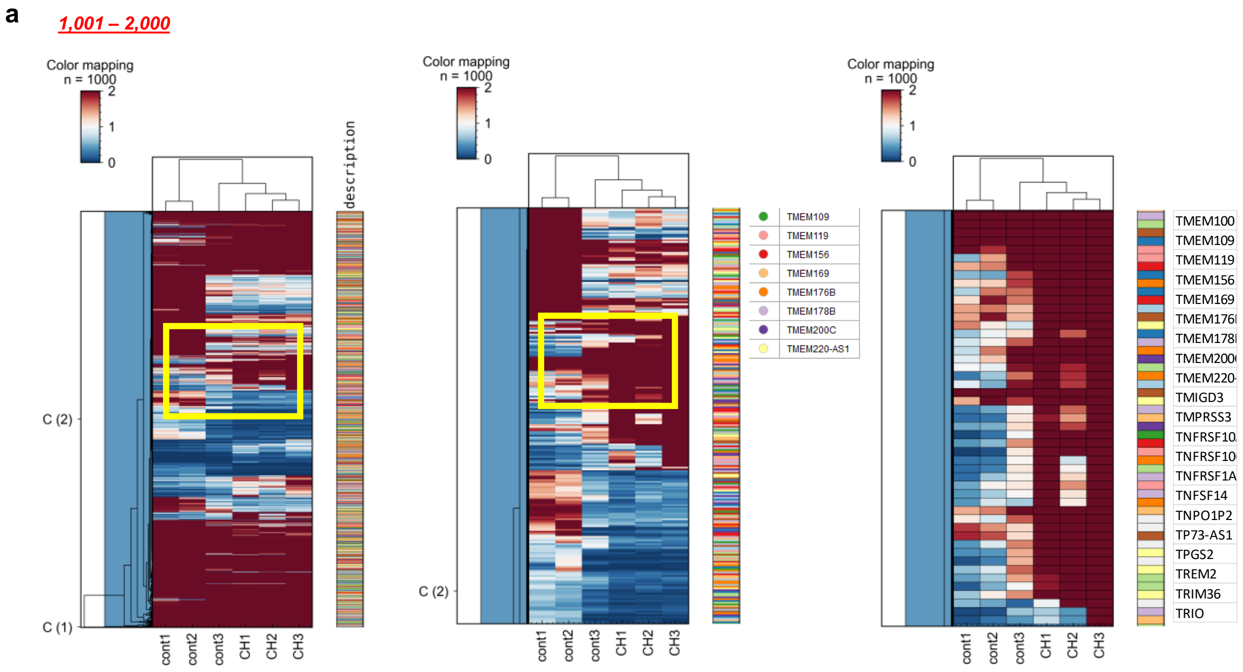

Figure S15. Transcriptomic profiling of the caudate nucleus in elderly individuals with chronic hydrocephalus (CH) versus age-matched controls.

(A) **Hierarchical clustering heatmap** of the top 1,000 differentially expressed genes (color scale: red = upregulated, blue = downregulated; n = 1000). Samples segregate into two major clusters (C1: controls; C2: chronic hydrocephalus), indicating distinct transcriptomic signatures between groups.

(B) **Gene expression pattern plots** showing representative gene clusters (C0–C7) derived from k-means clustering of normalized RNA-seq data. Each cluster exhibits characteristic expression trajectories distinguishing CH (red) from control (blue) samples.

(C) **Heatmap of selected xenobiotic metabolism–associated genes** (e.g., *PDK4, TTPA, NNMT, SMOX, SERPINE1, ADFP*) highlighting coordinated upregulation in CH samples.

(D) **Gene Set Enrichment Analysis (GSEA)** for the *HALLMARK_XENOBIOTIC_METABOLISM* pathway shows significant enrichment in CH (positive enrichment score, ES > 0.5), consistent with metabolic reprogramming and altered detoxification activity.

(E) **Random enrichment score distribution** plot confirming that CH samples (magenta) display positive ES values relative to controls (blue), supporting pathway-level activation.

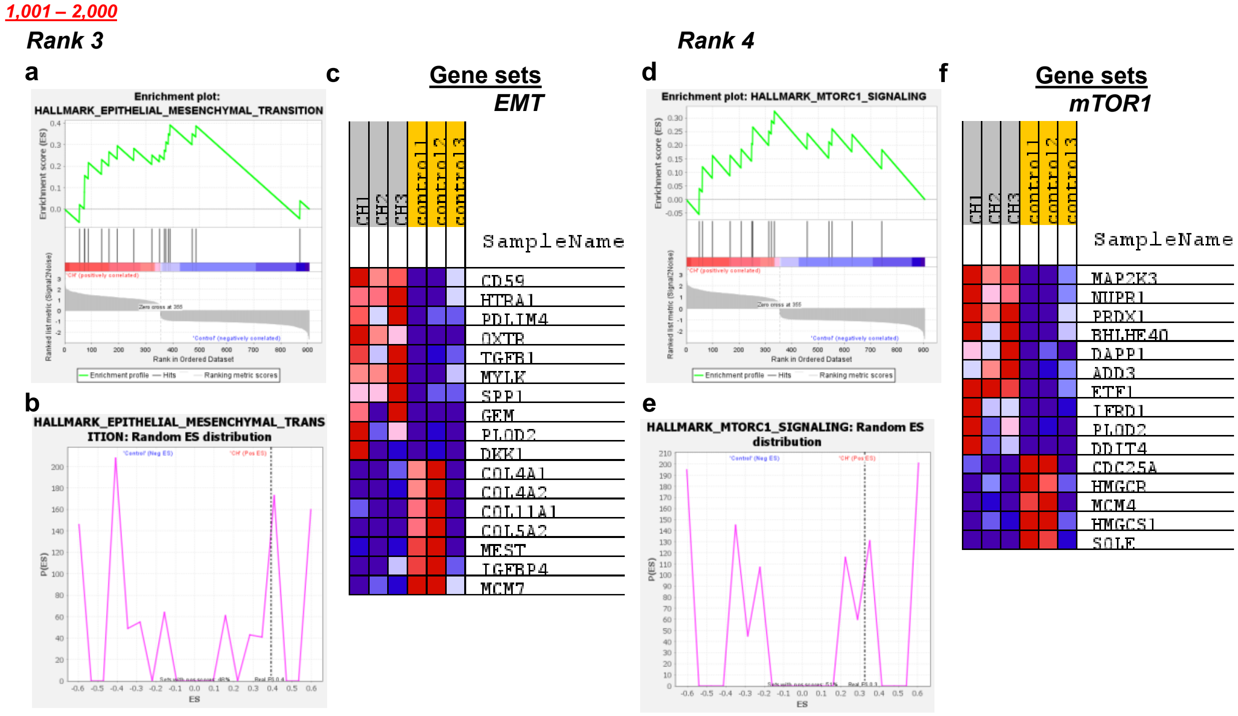

Figure S16. Gene Set Enrichment Analysis (GSEA) of epithelial–mesenchymal transition and mTORC1 signaling pathways using ranked genes 1,001–2,000.

(a) GSEA enrichment plot for the **HALLMARK_EPITHELIAL_MESENCHYMAL_TRANSITION** gene set based on differential expression ranks 1,001–2,000, demonstrating positive enrichment in the CH group relative to controls.

(b) **Permutation-based random enrichment score (ES) distribution** for the EMT gene set, with the observed ES indicated by a dashed vertical line.

(c) **Heatmap of leading-edge genes** contributing to EMT signaling within this ranked interval, showing coordinated regulation of extracellular matrix, cell adhesion, and mesenchymal-associated genes in CH samples compared with controls.

(d) GSEA enrichment plot for the **HALLMARK_MTORC1_SIGNALING** gene set derived from ranked genes 1,001–2,000, indicating enrichment in the CH group.

(e) **Random ES distribution** for the mTORC1 signaling gene set obtained by permutation testing.

(f) **Heatmap of leading-edge genes** associated with mTORC1 signaling, highlighting altered metabolic, translational, and cell-cycle–related transcriptional programs in CH samples within this gene rank window.

a

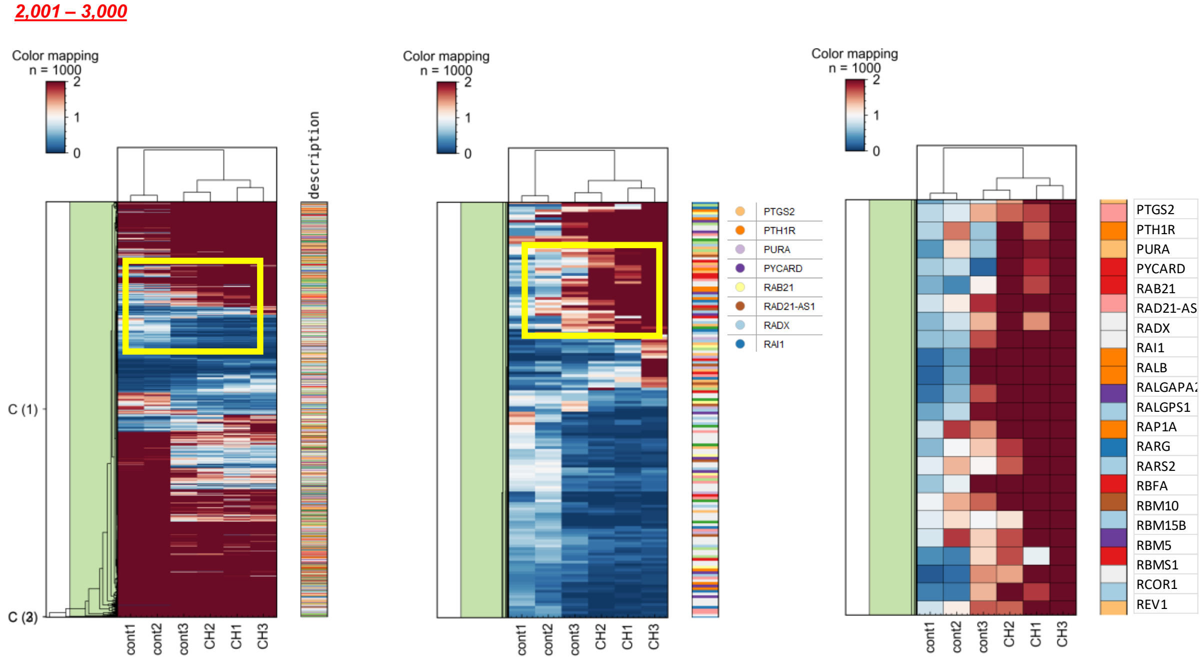

Figure S17. Hierarchical clustering of the 2,001–3,000 ranked variable transcripts revealing a secondary disease-associated gene program in chronic hydrocephalus. Heatmaps depict unsupervised hierarchical clustering of the third tier of transcriptomic variance (genes ranked 2,001–3,000 by variability) derived from bulk RNA-sequencing of the human caudate nucleus. Columns represent individual samples from chronic hydrocephalus (CH1–CH3) and age-matched neurologically normal controls (cont1–cont3), and rows represent genes scaled by Z-score expression. Dendrograms illustrate sample- and gene-level clustering, identifying two major co-expression clusters (C1 and C2). Yellow boxes highlight a prominent gene subcluster that exhibits coordinated upregulation in CH relative to controls, representing a secondary disease-associated transcriptional program distinct from the top 1,000 most variable genes. Selected representative genes within this module are indicated in the legend (e.g., *PTGS2, PTH1R, PYCRD, RAD21-AS1,* and *RADX*), suggesting involvement of inflammatory signaling, stress response, and DNA damage–associated pathways. The rightmost panels show an expanded view of representative genes from the highlighted module, demonstrating consistent directionality of expression changes across CH samples. These data indicate that transcripts ranked 2,001–3,000 capture additional coordinated molecular processes that contribute to chronic hydrocephalus–associated transcriptional divergence beyond the primary gene programs.

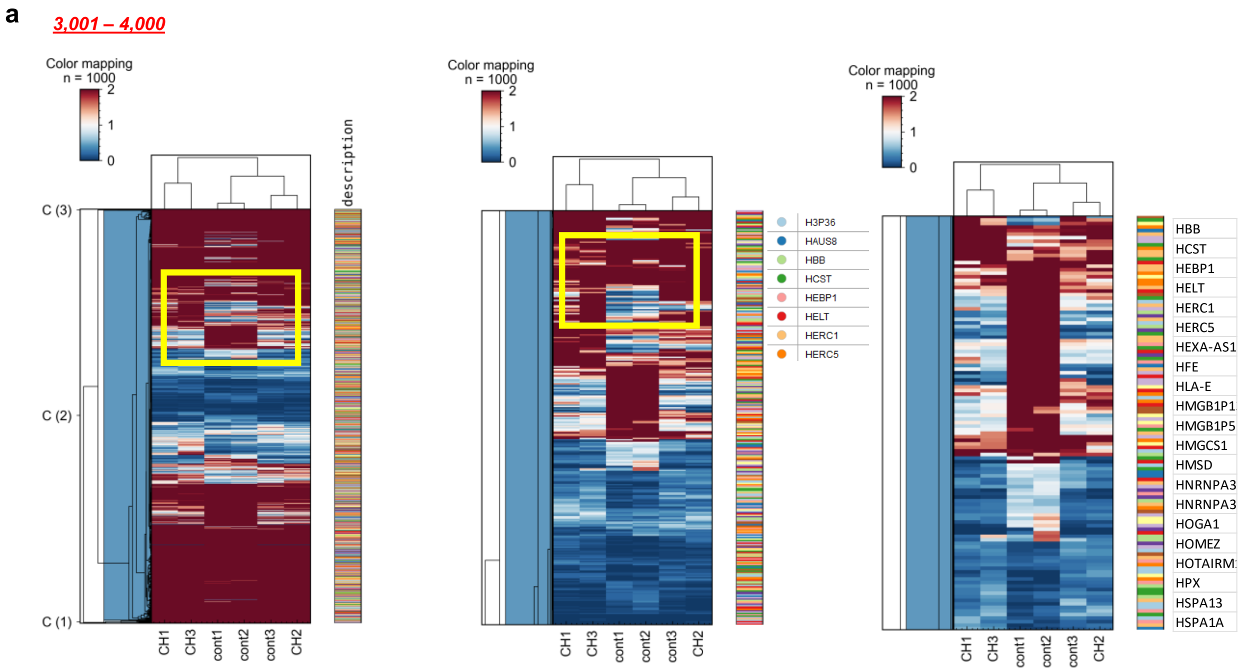

Figure S18. Hierarchical clustering of transcripts ranked 3,001–4,000 reveals a tertiary gene module enriched for hemoglobin- and interferon-associated signatures in chronic hydrocephalus.

Heatmaps show unsupervised hierarchical clustering of the fourth tier of transcriptomic variance (genes ranked 3,001–4,000 by variability) derived from bulk RNA-sequencing of human caudate nucleus tissue. Columns correspond to chronic hydrocephalus samples (CH1–CH3) and age-matched neurologically normal controls (cont1–cont3), and rows represent genes normalized by Z-score expression. Gene-level dendrograms identify three major co-expression clusters (C1–C3), indicating increased modular complexity compared with higher-ranked gene sets. Yellow boxes highlight a prominent subcluster within C3 that demonstrates coordinated upregulation in CH relative to controls. Representative genes within this module include hemoglobin-related transcripts (*HBB, HBP1, H3P36*) and interferon-stimulated or antiviral response genes (*HERC1, HERC5, HAUS8*), suggesting activation of erythroid-like, hypoxia-associated, and innate immune pathways in the diseased caudate nucleus. The rightmost panels provide an expanded view of representative genes from the highlighted cluster, illustrating consistent expression patterns across CH samples. These findings indicate that transcripts ranked 3,001–4,000 capture tertiary disease-associated programs linked to oxygen handling, stress adaptation, and immune signaling, further reinforcing the multi-layered nature of chronic hydrocephalus–associated transcriptional remodeling.

3,001 – 4,000

Rank 3

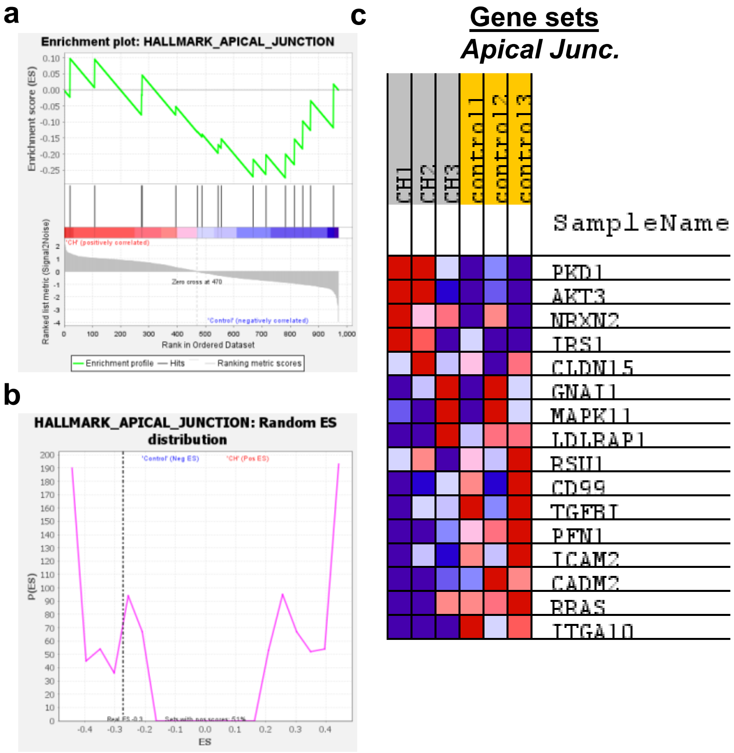

Figure S19. Gene Set Enrichment Analysis (GSEA) of apical junction signaling using ranked genes 3,001–4,000.

(a) GSEA enrichment plot for the **HALLMARK_APICAL_JUNCTION** gene set based on differential expression ranks 3,001–4,000, illustrating enrichment patterns in the CH group relative to controls.

(b) **Permutation-based random enrichment score (ES) distribution** for the apical junction gene set, with the observed ES indicated by a dashed vertical line.

(c) **Heatmap of leading-edge genes** contributing to apical junction signaling within this ranked interval, highlighting coordinated regulation of cell–cell adhesion, cytoskeletal organization, and junctional integrity genes across CH and control samples.

Normalized gene expression values are displayed using a red–blue color scale (red, higher expression; blue, lower expression). Vertical bars in enrichment plots indicate the positions of gene set members within the ordered gene list.

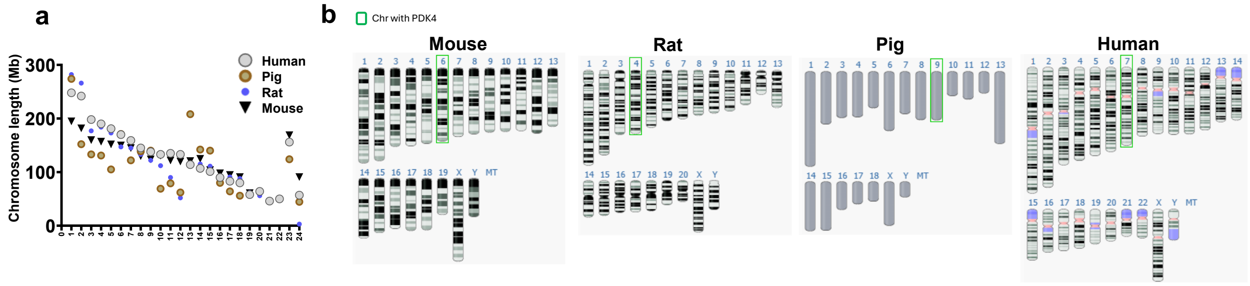

Figure S20. Comparative Chromosomal Architecture and Genomic Loci of PDK4 across Species.

(a) Chromosome length distributions in mouse, rat, pig, and human, illustrating species-specific differences in chromosomal architecture that influence gene positioning and mutation susceptibility.

(b) Relative chromosome sizes across species. Schematic karyotypes of mouse, rat, pig, and human emphasizing proportional chromosome lengths; illustrations were independently redrawn based on publicly available genome annotations. Locations of PDK4 (green) are highlighted to contextualize their genomic positioning within each karyotype.

Table S1 Inclusion criteria: human postmortem tissues from the Neurobiobank

Criteria	Tissue	RNA integrity number (RIN)	Age	Type	Sex	HIV*	HBSAG**	PMInterval***
Include	Cortex, caudate nucleus, cerebellum	7 – 10	≥ 65 yr	Frozen	Male & female	Negative	Negative	< 36 hr
Exclude	elsewhere	<7	< 65 yr	Fixed	-	Positive	Positive	≥ 36 hr

* Human immunodeficiency virus; ** Hepatitis B Surface Antigen Test; *** Postmortem interval

Table S2 Postmortem specimen information

Numbering	Subject ID	Age (years)	Disorder	Sex	Race	Medical History	RNA-seq session #	RT-PCR ?
1	5219	76	Unaffected Control	Female	White		2	Yes
2	4921	73	Unaffected Control	Female	White	*	2	Yes
3	4789	72	Unaffected Control	Female	White		2	Yes
4	42055	85	Unaffected Control	Male	White		1	Yes
5	5671	78	Unaffected Control	Male	White		-	Yes
6	3642	88	Unaffected Control	Female	White		1	Yes
7	994765	86	Hydrocephalus	Male	White	**	1	Yes
8	5605	76	Hydrocephalus	Female	White		1	Yes
9	4922	79	Hydrocephalus	Male	White	***	2	Yes
10	3924	79	Hydrocephalus	Female	White	§	2	Yes
11	3637	76	Hydrocephalus	Male	White	§§	2	Yes
12	21762	77	Hydrocephalus	Female	African American	§§§	-	Yes

* gall bladder problem, ** idiopathic, *** TBI, traumatic brain injury, § MS, Multiple Sclerosis, §§ Cerebral atherosclerosis, §§§ Vascular dementia.

Table S3 Primer sequences for *human* gene transcripts

**PDK4** (product size: 249 b); Exon 2<br>(upstream) TCAGCCTTCCCTTACACCAA<br>(downstream) ACAAGTCATCAAAGCCACACA	**HMOX1** (product size: 211 b); Exon 3<br>(upstream) GTCTTCGCCCCTGTCTACTT<br>(downstream) CAGACAGGTCACCCAGGTAG
**RelB** (product size: 154 b); Exon 1<br>(upstream) GATCGTCCACCAGACCGTG<br>(downstream) CTAAGGCCCCCAGCTCCG	**GAPDH** (product size: 201 b); Exon 6<br>(upstream) ACCCAGAAGACTGTGGATGG<br>(downstream) TTCTAGACGGCAGGTCAGGT
**TRPV4** (product size: 204 b); Exon 2<br>(upstream) ATCTGTTTGAGGGGGAGGAT<br>(downstream) AGTCCATGGGTGCTTTCTTG	**HBA2** (product size: 157 b); Exon 3<br>(upstream) ACAAGTTCCTGGCTTCTGTG<br>(downstream) TGCCCACTCAGACTTTATTCAA

Table S4 Mutability Analyses through Two Factors[1-8] over Human Chromosomes

Human	Chr #	Gene Loc	Telom.	Proximity, Mb	A	T	A+T (%)	Full length (b)
PDK4	7	95	158	63	30	31	61	3601
HMOX1	22	35	50	15	20	24	44	1554
Relb	19	45	58	13	17	19	36	2258
TRPV4	12	109	133	24	18	23	41	3228
HBA2	16	0.1	0	0.1	17	19	36	576
APP	21	26	45	19	27	25	52	3583
MAPT	17	45	83	38	22	23	45	5639
SNCA	4	89	190	101	30	33	63	3177

* Chr, chromosome; Loc, locus; Telom, telomere locus; A, adenine; T, thymine; b, (nucleotide) base

Table S5 Mutability Analyses through Two Factors[1-8] over Porcine Chromosomes

Pig	Chr #	Gene Loc	Telom.	Proximity, Mb	A	T	A+T (%)	Full length (b)
PDK4	9	75	139	64	31	31	62	3590
HMOX1	NA	1.95	1.98	0.03	19	22	41	1552
Relb	6	51	170	119	17	19	36	2281
TRPV4	14	41	141	100	20	22	42	2616
HBA2	NA	NA	NA	NA	NA	NA	NA	NA
APP	13	189	207	18	27	24	51	3051
MAPT	12	17	61	44	21	22	43	5063
SNCA	8	129	138	9	28	27	55	982

* Chr, chromosome; Loc, locus; Telom, telomere locus; A, adenine; T, thymine; b, (nucleotide) base; NA, not available

Table S6 Mutability Analyses through Two Factors[1-8] over Rat Chromosomes

Rat	Chr #	Gene Loc	Telom.	Proximity, Mb	A	T	A+T (%)	Full length (b)
PDK4	4	34	184	150	25	25	50	1425
HMOX1	19	13	74	61	24	25	49	1600
Relb	1	88	270	182	19	22	41	2160
TRPV4	12	47	52	5	20	23	43	3211
HBA2	10	15	107	92	22	23	45	548
APP	11	37	99	62	26	21	47	2340
MAPT	10	89	107	18	24	24	48	5149
SNCA	4	91	184	93	28	27	55	1150

* Chr, chromosome; Loc, locus; Telom, telomere locus; A, adenine; T, thymine; b, (nucleotide) base

Table S7 Mutability Analyses through Two Factors[1-8] over Murine Chromosomes

Mouse	Chr #	Gene Loc	Telom.	Proximity, Mb	A	T	A+T (%)	Full length (b)
PDK4	6	5.4	149,500k	144	26	30	56	3453
HMOX1	8	75	130	55	25	24	49	1634
Relb	7	19	144	125	19	21	40	2193
TRPV4	5	114	151	37	20	22	42	3247
HBA2	11	32	122	90	23	24	47	587
APP	16	84	98	14	27	23	50	3152
MAPT	104	104	121	17	24	25	49	5164
SNCA	6	60	149	89	29	28	57	1208

* Chr, chromosome; Loc, locus; Telom, telomere locus; A, adenine; T, thymine; b, (nucleotide) base

Table S8 RNA-seq Quality Metric (RIN)

Sample name	RIN	Sample group (diagnosis)	RNA-seq batch
CN3	7	Control	2 (summer*)
C5	8	Control	2 (summer)
C3	8.8	Control	2 (summer)
CN7	>6	Control	1 (winter **)
CN8	>6	Control	1 (winter)
CH4	7	Hydrocephalus	2 (summer)
CH5	6.9	Hydrocephalus	2 (summer)
CH6	6.5	Hydrocephalus	2 (summer)
72CH	>6	Hydrocephalus	1 (winter)
902CH	>6	Hydrocephalus	1 (winter)

*** 8/17/2023, **1/5/2023**

Supplementary Methods for Heat Map Ranking:

1. Data Pre-processing and Ranking

The ranking of genes for heatmap visualization is primarily driven by their Differential Expression Analysis (DEA) significance.

- Differential Expression (DEGs): Transcripts are ranked based on a combination of Fold-Change (FC) and p-value (typically adjusted via the Benjamini-Hochberg procedure).
- Selection of Scopes: This ranking allows for the systematic selection of "Top" gene sets (e.g., Top 25, 100, or 1,000 DEGs), ensuring that the visualization focuses on the most statistically robust drivers of the chronic hydrocephalus (CH) phenotype.

2. Hierarchical Clustering (Row and Column Ranking)

Within the heatmap, the relative position (ranking) of genes (rows) and samples (columns) is determined by hierarchical clustering:

- Distance Metric: InstantClue typically utilizes Euclidean distance or Pearson correlation to calculate the similarity between expression profiles.
- Linkage Method: The software applies Ward's linkage or Average linkage to construct the dendrogram.
- Visual Result: This ranks genes with similar expression trajectories together, creating the clusters C(1) and C(2). At the global scale (n=40,480), this hierarchical ranking reflects aggregate similarity, whereas at high-resolution (Top 25), it isolates the core pathological drivers like *PDK4*.

3. K-means Trajectory Ranking (k=8)

To complement the hierarchical ranking, we employed K-means clustering to categorize genes into eight distinct "expression programs".

- Centroid Optimization: This system ranks and assigns each gene to a cluster whose centroid (mean expression profile) is most similar to the gene's own normalized trajectory.
- Magnitude Stability: As shown in our results, this dual-ranking system (hierarchical + K-means) consistently places *PDK4* in high-magnitude, upward-trending clusters (C2 or C3), validating its position as a dominant disease-associated driver regardless of the total gene count analyzed.

4. Normalization and Scaling

Finally, the "Color Mapping" ranking (0 to 2) is based on Z-score standardization. This scales the expression values so the mean is 0 and the standard deviation is 1, allowing for a standardized visual comparison of genes across a wide range of absolute FPKM magnitudes.

References for supplementary material

1 Cunningham, A. *et al.* NFkappaB1: a common biomarker linking Alzheimer's and Parkinson's disease pathology. *Front Neurosci* **19**, 1589857 (2025). https://doi.org/10.3389/fnins.2025.1589857

2 Barrett, E. *et al.* Reduced GLP-1R availability in the caudate nucleus with Alzheimer's disease. *Front Aging Neurosci* **16**, 1350239 (2024). https://doi.org/10.3389/fnagi.2024.1350239

3 McKnight, I. *et al.* Mutability of druggable kinases and pro-inflammatory cytokines by their proximity to telomeres and A+T content. *PLoS One* **18**, e0283470 (2023). https://doi.org/10.1371/journal.pone.0283470

4 Hart, M. *et al.* X-linked hydrocephalus genes: Their proximity to telomeres and high A + T content compared to Parkinson's disease. *Exp Neurol* **366**, 114433 (2023). https://doi.org/10.1016/j.expneurol.2023.114433

5 White, H. *et al.* TRPV4 mRNA is elevated in the caudate nucleus with NPH but not in Alzheimer's disease. *Front Genet* **13**, 936151 (2022). https://doi.org/10.3389/fgene.2022.936151

6 Raines, R. *et al.* Drug-Targeted Genomes: Mutability of Ion Channels and GPCRs. *Biomedicines* **10** (2022). https://doi.org/10.3390/biomedicines10030594

7 McKnight, I., Hart, C., Park, I. H. & Shim, J. W. Genes causing congenital hydrocephalus: Their chromosomal characteristics of telomere proximity and DNA compositions. *Exp Neurol* **335**, 113523 (2021). https://doi.org/10.1016/j.expneurol.2020.113523

8 Lucas, H. B. *et al.* Factors Associated with Mutations: Their Matching Rates to Cardiovascular and Neurological Diseases. *Int J Mol Sci* **22** (2021). https://doi.org/10.3390/ijms22105057
